# A Prospective, Randomized, Open-Label Trial of Early versus Late Favipiravir Therapy in Hospitalized Patients with COVID-19

**DOI:** 10.1128/AAC.01897-20

**Published:** 2020-11-17

**Authors:** Yohei Doi, Masaya Hibino, Ryota Hase, Michiko Yamamoto, Yu Kasamatsu, Masahiro Hirose, Yoshikazu Mutoh, Yoshito Homma, Masaki Terada, Taku Ogawa, Fumihiro Kashizaki, Toshihiko Yokoyama, Hayato Koba, Hideki Kasahara, Kazuhisa Yokota, Hideaki Kato, Junichi Yoshida, Toshiyuki Kita, Yasuyuki Kato, Tadashi Kamio, Nobuhiro Kodama, Yujiro Uchida, Nobuhiro Ikeda, Masahiro Shinoda, Atsushi Nakagawa, Hiroki Nakatsumi, Tomoya Horiguchi, Mitsunaga Iwata, Akifumi Matsuyama, Sumi Banno, Takenao Koseki, Mayumi Teramachi, Masami Miyata, Shigeru Tajima, Takahiro Maeki, Eri Nakayama, Satoshi Taniguchi, Chang Kweng Lim, Masayuki Saijo, Takumi Imai, Hisako Yoshida, Daijiro Kabata, Ayumi Shintani, Yukio Yuzawa, Masashi Kondo

**Affiliations:** a Departments of Microbiology and Infectious Diseases, Fujita Health University School of Medicine, Toyoake, Aichi, Japan; b Division of Infectious Diseases, University of Pittsburgh School of Medicine, Pittsburgh, Pennsylvania, USA; c Department of Emergency and General Internal Medicine, Fujita Health University School of Medicine, Toyoake, Aichi, Japan; d Department of Infectious Diseases, Japanese Red Cross Narita Hospital, Narita, Chiba, Japan; e Department of Respiratory Medicine, Sagamihara Kyodo Hospital, Sagamihara, Kanagawa, Japan; f Infection Control and Clinical Laboratory, Kyoto Prefectural University of Medicine, Kyoto, Kyoto, Japan; g Department of Respiratory Medicine, Fujita Health University Bantane Hospital, Nagoya, Aichi, Japan; h Department of Infectious Diseases, Tosei General Hospital, Seto, Aichi, Japan; i Department of Respiratory Medicine, Ehime Prefectural Central Hospital, Matsuyama, Ehime, Japan; j Department of Respiratory Medicine, Saiseikai Niigata Hospital, Niigata, Niigata, Japan; k Center for Infectious Diseases, Nara Medical University, Kashihara, Nara, Japan; l Department of Respiratory Medicine, Isehara Kyodo Hospital, Isehara, Kanagawa, Japan; m Department of Respiratory Medicine, Japanese Red Cross Nagoya Daiichi Hospital, Nagoya, Aichi, Japan; n Department of Respiratory Medicine, Komatsu Municipal Hospital, Komatsu, Ishikawa, Japan; o Department of Rheumatology, NTT East Sapporo Hospital, Sapporo, Hokkaido, Japan; p Department of Infectious Diseases, Tokyo Metropolitan Health and Medical Corporation Ebara Hospital, Ohta, Tokyo, Japan; q Infection Prevention and Control Department, Yokohama City University Hospital, Yokohama, Kanagawa, Japan; r Department of Chest Surgery, Shimonoseki City Hospital, Shimonoseki, Yamaguchi, Japan; s Department of Respiratory Medicine, National Hospital Organization Kanazawa Medical Center, Kanazawa, Ishikawa, Japan; t Department of Infectious Diseases, International University of Health and Welfare Narita Hospital, Narita, Chiba, Japan; u Department of Intensive Care, Shonan Kamakura General Hospital, Kamakura, Kanagawa, Japan; v Department of General Internal Medicine, Fukuoka Tokushukai Hospital, Kasuga, Fukuoka, Japan; w Department of General Medicine, Kitakyushu Municipal Medical Center, Kitakyushu, Fukuoka, Japan; x Department of General Internal Medicine, Eiju General Hospital, Taito, Tokyo, Japan; y Department of Respiratory Medicine, Tokyo Shinagawa Hospital, Shinagawa, Tokyo, Japan; z Department of Respiratory Medicine, Kobe City Medical Center General Hospital, Kobe, Hyogo, Japan; aa Department of Respiratory Medicine, Ishikawa Prefectural Central Hospital, Kanazawa, Ishikawa, Japan; bb Department of Respiratory Medicine, Fujita Health University School of Medicine, Toyoake, Aichi, Japan; cc Department of Regenerative Medicine and Stem Cell Biology, Fujita Health University School of Medicine, Toyoake, Aichi, Japan; dd Center for Clinical Trial and Research Support, Fujita Health University School of Medicine, Toyoake, Aichi, Japan; ee Department of Clinical Pharmacy, Fujita Health University School of Medicine, Toyoake, Aichi, Japan; ff Department of Virology I, National Institute of Infectious Diseases, Shinjuku, Tokyo, Japan; gg Department of Medical Statistics, Osaka City University Graduate School of Medicine, Osaka, Osaka, Japan; hh Department of Nephrology, Fujita Health University School of Medicine, Toyoake, Aichi, Japan

**Keywords:** COVID-19, antiviral therapy, randomized clinical trial, Avigan

## Abstract

Favipiravir is an oral broad-spectrum inhibitor of viral RNA-dependent RNA polymerase that is approved for treatment of influenza in Japan. We conducted a prospective, randomized, open-label, multicenter trial of favipiravir for the treatment of COVID-19 at 25 hospitals across Japan. Eligible patients were adolescents and adults admitted with COVID-19 who were asymptomatic or mildly ill and had an Eastern Cooperative Oncology Group (ECOG) performance status of 0 or 1. Patients were randomly assigned at a 1:1 ratio to early or late favipiravir therapy (in the latter case, the same regimen starting on day 6 instead of day 1). The primary endpoint was viral clearance by day 6. The secondary endpoint was change in viral load by day 6. Exploratory endpoints included time to defervescence and resolution of symptoms. Eighty-nine patients were enrolled, of whom 69 were virologically evaluable. Viral clearance occurred within 6 days in 66.7% and 56.1% of the early and late treatment groups (adjusted hazard ratio [aHR], 1.42; 95% confidence interval [95% CI], 0.76 to 2.62). Of 30 patients who had a fever (≥37.5°C) on day 1, times to defervescence were 2.1 days and 3.2 days in the early and late treatment groups (aHR, 1.88; 95% CI, 0.81 to 4.35). During therapy, 84.1% developed transient hyperuricemia. Favipiravir did not significantly improve viral clearance as measured by reverse transcription-PCR (RT-PCR) by day 6 but was associated with numerical reduction in time to defervescence. Neither disease progression nor death occurred in any of the patients in either treatment group during the 28-day participation. (This study has been registered with the Japan Registry of Clinical Trials under number jRCTs041190120.)

## Abstract (precis)

Favipiravir is an oral broad-spectrum inhibitor of viral RNA-dependent RNA polymerase that is approved for treatment of influenza in Japan. We conducted a prospective, randomized, open-label, multicenter trial of favipiravir for the treatment of COVID-19 at 25 hospitals across Japan. Eligible patients were adolescents and adults admitted with COVID-19 who were asymptomatic or mildly ill and had an Eastern Cooperative Oncology Group (ECOG) performance status of 0 or 1. Patients were randomly assigned at a 1:1 ratio to early or late favipiravir therapy (in the latter case, the same regimen starting on day 6 instead of day 1).

## INTRODUCTION

The new coronavirus severe acute respiratory syndrome coronavirus 2 (SARS-CoV-2) has spread throughout the globe since late 2019 and caused an unprecedented pandemic. The virus has infected at least 28 million people and caused over 900,000 deaths due to COVID-19. While the majority of those infected with SARS-CoV-2 develop mild, self-limiting disease, with even some remaining fully asymptomatic throughout the course, some patients progress to severe pneumonia, multiorgan failure, and death (1).

Favipiravir is an oral, broad-spectrum inhibitor of viral RNA-dependent RNA polymerase which also elicits viral mutagenesis (2). Its mechanism of action is selective inhibition of viral RNA polymerase *in vivo* by its triphosphorylated derivative (T-705RTP), which translates to broad-spectrum inhibition of RNA viruses (3). It is currently approved in Japan for the treatment of emerging and reemerging influenza virus infection for which other anti-influenza drugs are ineffective or not sufficiently effective (4). Favipiravir has *in vitro* activity against SARS-CoV-2, and a nonrandomized study conducted in China has shown significantly shorter time to viral clearance among patients with mild to moderate COVID-19 who were treated with favipiravir and interferon alpha than in those treated with lopinavir-ritonavir and interferon alpha (5). Interim results of a pilot multicenter, randomized trial conducted in Russia have also shown a significantly higher rate of viral clearance on the fifth day among hospitalized COVID-19 patients who were randomized to avifavir, favipiravir that was resynthesized in Russia, compared with standard of care (6).

The objective of this study was to evaluate the efficacy of favipiravir in achieving viral clearance for patients with asymptomatic to mildly symptomatic COVID-19 infection and clinical improvement among those showing symptoms.

## RESULTS

### Patients.

**FIG 1:**
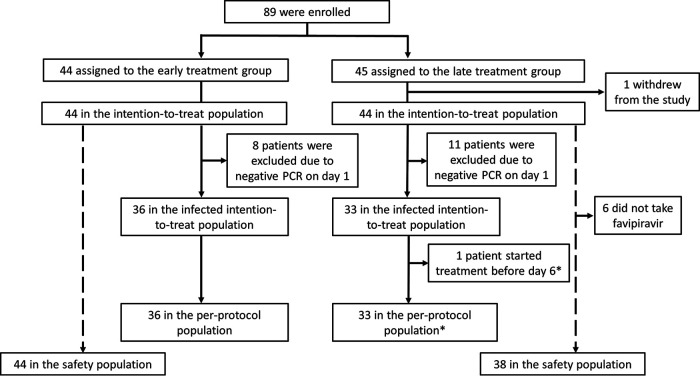
Patient enrollment and treatment assignment. *, per-protocol population analysis excluded all observations after the time of the protocol violation.

A total of 89 patients with laboratory-confirmed SARS-CoV-2 infection were randomized: 44 were assigned to the early treatment group and 45 to the late treatment group. One patient withdrew from the study immediately after randomization, leaving 88 patients who had any study-related data and comprised the intention-to-treat (ITT) population. The infected ITT population, defined for the primary outcome analysis of the viral clearance, consisted of 36 and 33 patients in the early and late treatment groups after excluding 8 and 11 patients whose reverse transcription-PCR (RT-PCR) result on the first day was already negative. Finally, the safety population included 44 patients in the early treatment group and 38 patients in the late treatment group after excluding 7 patients who did not take any favipiravir dose (Fig. 1). The day of randomization was day 1 in 86 patients. For the remaining 3 patients (2 in the early treatment group and 1 in the late treatment group), day 1 was the day following randomization since randomization took place in the evening.

The median age was 50.0 years (interquartile range [IQR], 38.0 to 64.5 years), and 54 (61.4%) patients were men in the ITT population (*n* = 88). A total of 32 patients (36.4%) had a body temperature of 37.5°C or greater at enrollment (Table 1). The median interval between the first positive RT-PCR and randomization was 4.0 days (IQR, 2.5 to 5.0 days), and the median interval between onset of fever and randomization was 7.0 days (IQR, 5.0 to 10.0 days) (Table 1).

**TABLE 1:** Baseline demographics and clinical characteristics of COVID-19 patients in the ITT population

Demographic and clinical characteristics^*a*^	Value for:		
	Early treatment (*n* = 44)	Late treatment (*n* = 44)	ITT population (*n* = 88)
Age, median (ICR), yrs	48.0 (34.5, 68.0)	51.0 (39.5, 62.0)	50.0 (38.0, 64.5)
Sex, no. (%)			
Male	23 (52.3)	31 (70.5)	54 (61.4)
Female	21 (47.7)	13 (29.5)	34 (38.6)
Body mass index, median (ICR)	22.5 (20.5, 24.9)	23.6 (21.8, 26.0)	23.4 (20.8, 25.9)
Coexisting diseases, no. (%)	15 (34.1)	19 (43.2)	34 (39)
Symptomatic, no. (%)	30 (68)	31 (70)	61 (69)
Subjective fever, no. (%)	14 (31.8)	18 (40.9)	32 (36)
Cough, no. (%)	19 (43.2)	13 (29.5)	32 (36)
Dyspnea, no. (%)	3 (6.8)	5 (11.4)	8 (9)
Myalgia or arthralgia, no. (%)	3 (6.8)	3 (6.8)	6 (7)
Need for supplemental oxygen at randomization, no (%)	1 (2.3)	2 (4.6)	3 (3.4)
Time between fever onset and randomization, median (IQR), days^*b*^	7.0 (5.5, 10.0)	8.0 (5.0, 10.0)	7.0 (5.0, 10.0)
Time between positive RT-PCR and randomization, median (IQR), days	4.0 (3.0, 5.0)	4.0 (2.0, 5.0)	4.0 (2.5, 5.0)
Laboratory values			
SpO_2_, median, %	96.0 (95.0, 97.0)	96.0 (95.0, 97.0)	96.0 (95.0, 97.0)
Body temp, median (IQR), °C	37.1 (36.7, 38.0)	37.0 (36.7, 38.0)	37.1 (36.7, 38.0)
Systolic blood pressure, median (IQR), mm Hg	124.0 (117.0, 136.0)	121.0 (113.5, 128.0)	122.0 (115.5, 133.5)
Heart rate, median (IQR), /min	78.0 (75.0, 84.5)	76.0 (66.5, 82.0)	77.0 (68.5, 84.0)
Respiratory rate, median (IQR), /min	16.0 (16.0, 21.0)	17.5 (16.0, 18.5)	17.0 (16.0, 20.0)
White blood cell count, median (IQR), cells/μl	4,4 (3.6, 5.8)	5.1 (4.0, 6.4)	4.8 (3.8, 6.0)
Platelet count, median (IQR), ×10^3^/μl	188.5 (152.5, 250.5)	210.0 (170.5, 260.0)	204 (163.0, 255.0)
CRP, median (IQR), mg/liter	1.1 (0.2, 3.9)	0.7 (0.1, 3.5)	0.8 (0.2, 3.8)
ALT, median (IQR), U/liter	19.5 (13.0, 36.5)	20.5 (16.0, 32.0)	20.0 (14.5, 35.5)
AST, median (IQR), U/liter	24.0 (18.0, 29.0)	23.5 (18.5, 31.0)	24.0 (18.0, 30.0)
Urea nitrogen, median (IQR), mg/dl	11.8 (10.0, 14.5)	12.7 (10.9, 16.1)	12.3 (10.5, 15.1)
Serum creatinine, median (IQR), mg/dl	0.8 (0.6, 0.9)	0.8 (0.7, 1.0)	0.8 (0.7, 0.9)
Uric acid, median (IQR), mg/dl	4.3 (3.3, 5.3)	4.8 (4.0, 6.1)	4.7 (3.6, 5.4)
Viral load (IQR), copies/ml	104.2 (102.5, 106.2)	104.8(102.2, 106.2)	104.4(102.5, 106.2)
No. (%) with concomitant therapy			
Systemic antibiotics	8 (18.2)	3 (6.8)	11 (12.5)
Antiviral agents other than favipiravir	0 (0.0)	0 (0.0)	0 (0.0)
Systemic corticosteroids	2 (4.5)	0 (0.0)	2 (2.3)
Antiplatelet/antithrombotic agents^*c*^	0 (0.0)	0 (0.0)	0 (0.0)

a CRP, C-reactive protein; ALT, alanine aminotransferase; AST, aspartate aminotransferase.

b *n* = 32 for the early treatment group; *n* = 28 for the late treatment group.

c Excludes therapy that patients were receiving prior to the diagnosis of COVID-19 and continued in hospital.

The two groups were similar overall in their demographic and clinical characteristics as well as baseline laboratory results, but there was an imbalance in the male-to-female ratio, with males accounting for 52.3% in the early treatment group and 70.5% in the late treatment group. This imbalance persisted in the infected ITT population (Table S1).

### Efficacy.

**FIG 2:**
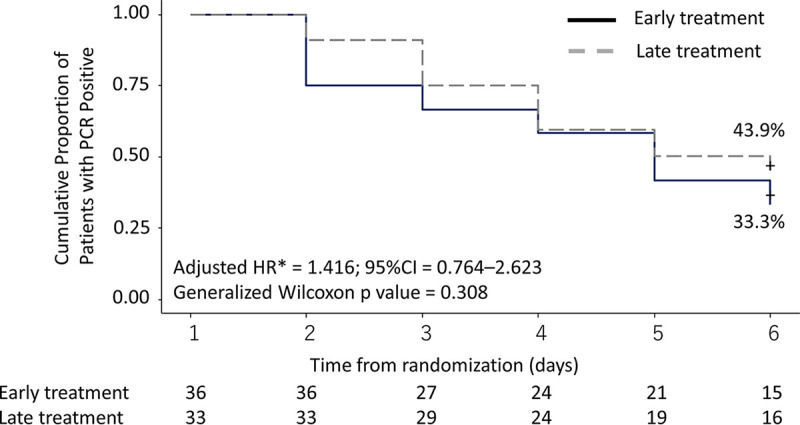
Viral clearance by day 6 among the infected intention-to-treat population. *, HR is adjusted for age and days between collection of the SARS-CoV-2-positive specimen and enrollment.

In the infected ITT population, the likelihood of viral clearance by day 6 was not significantly different between the early treatment and late treatment groups (Table 2). Viral clearance by day 6 was achieved in 66.7% (95% confidence interval [CI], 51.4 to 81.2) in the early treatment group and 56.1% (95% CI, 40.1 to 73.4) in the late treatment group (Fig. 2). The adjusted hazard ratio (aHR) of viral clearance by day 6 after adjusting for age and days from the first positive RT-PCR was 1.416 (95% CI, 0.764 to 2.623).

**TABLE 2:** Outcomes in the infected ITT and ITT populations

Population and parameter	Value for:		Effect estimate (95% CI)^*a*^
	Early treatment	Late treatment	
Infected ITT population	36	33	
Primary outcome			
SARS-CoV-2 clearance by day 6, %	66.7	56.1	HR = 1.416 (0.764–2.623)
Secondary outcomes			
SARS-CoV-2 clearance by day 10, %	86.1	83.1	HR = 1.271 (0.744–2.172)
50% logarithmic reduction in the SARS-CoV-2 viral load by day 6, %	94.4	78.8	OR = 4.750 (0.876–25.764)
Median time until SARS-CoV-2 clearance by local RT-PCR, days	12.8	17.8	HR = 1.416 (0.764–2.623)
*Post hoc* analysis			
Median time until hospital discharge, days	14.0	21.5	HR = 2.677 (1.672–4.285)
			
ITT population	44	44	
Exploratory outcomes			
Disease progression or death, %^*b*^	0.0	0.0	ND
Post hoc analysis			
Median time until hospital discharge, days	14.5	20.0	HR = 1.963 (1.331–2.894)
			
ITT population (only patients with fever on day 1)	16	14	
Exploratory outcomes			
Duration of fever of ≥37.5°C, days	2.1	3.2	HR = 1.880 (0.812–4.354)
Duration of fever of ≥37.0°C, days	2.5	3.2	HR = 1.428 (0.700–2.911)

a All effect estimates were adjusted for age and days between collection of the SARS-CoV-2-positive specimen and enrollment.

b Disease progression was defined as need for mechanical ventilation or intensive care unit (ICU) admission. ND, not determined.

The proportions of patients with 50% logarithmic reduction in the SARS-CoV-2 viral load by day 6 in the infected ITT population were 94.4% and 78.8%, respectively (adjusted odds ratio [aOR], 4.750; 95% CI, 0.876 to 25.764). The change in viral load in the infected ITT population was more negative in the early treatment group than the late treatment group, but the difference was not statistically significant (Fig. S2).

**FIG 3:**
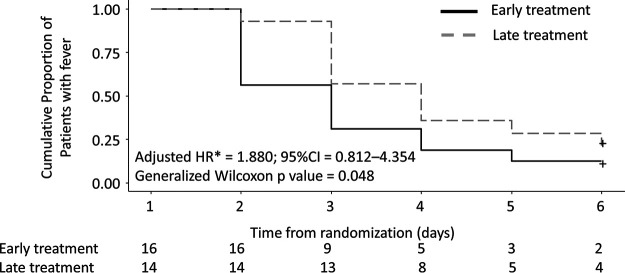
Time to defervescence (<37.5°C) among the intention-to-treat population. Only those with temperatures of ≥37.5°C on day 1 are included. *, HR is adjusted for age and days between collection of the SARS-CoV-2-positive specimen and enrollment.

Mean times to defervescence defined by the highest temperature of a given day being below 37.5°C were 2.1 days (95% CI, 1.421 to 2.846) in the early treatment group and 3.2 days (95% CI, 2.390 to 3.918) in the late treatment group (aHR, 1.880; 95% CI, 0.812 to 4.354) in the ITT population (Fig. 3). Mean times to defervescence defined by the highest temperature of a given day being below 37.0°C were 2.5 days (95% CI, 1.830 to 3.217) in the early treatment group and 3.2 days (95% CI, 2.591 to 3.790) in the late treatment group (aHR, 1.428; 95% CI, 0.700 to 2.911) in the ITT population. The change in body temperature in the ITT population was more negative in the early treatment group than the late treatment group, with a statistically significant difference on the second day (Fig. S2). The changes in clinical symptoms in the ITT population, either with a cumulative number of 4 major symptoms (subjective fever, cough, dyspnea, and myalgia/arthralgia) or all 13 symptoms recorded (the 4 symptoms plus sore throat, headache, chills/diaphoresis, fatigue, disturbance of consciousness, rhinorrhea, chest pain, diarrhea, and nausea/vomiting) were comparable between the two groups (Fig. S3 and S4). Neither disease progression nor death occurred in any of the patients in either treatment group during the 28-day participation.

Prespecified subgroups for the primary outcome analysis included age of ≥65 years, viral load on the first day, time from the first positive RT-PCR, and presence of any symptom on the first day. Viral clearance by the sixth day was significantly more likely to occur in the early treatment group than the late treatment group for patients with age ≥65 (8/9 [88.9%] versus 0/5 [0%]). However, there was an imbalance in the initial viral load between the two groups despite stratified randomization (10^4.3^ copies/ml versus 10^7.7^ copies/ml). The early treatment group was also more likely to achieve viral clearance than the late treatment group for patients who were randomized ≥4 days (median) after the first positive RT-PCR (HR, 2.829, 95% CI, 1.198 to 6.683).

### Safety.

A total of 144 adverse events were reported among the 82 patients in the safety population, consisting of patients who received at least one dose of favipiravir. The most common was hyperuricemia, which occurred in 69/82 (84.1%). Of 32 patients who had serum uric acid level determined on the 16th or 28th day (early treatment group) or on the 28th day (late treatment group), 24 patients had the level normalized to below 7 mg/dl, with the highest being 8.8 mg/dl. Other reported adverse events included serum triglyceride elevation (9/82 [11.0%]) and serum alanine aminotransferase elevation (7/82 [8.5%]).

## DISCUSSION

In this randomized clinical trial of asymptomatic to mild COVID-19 patients, there was no significant difference in the rate of SARS-CoV-2 clearance by day 6 between those who received favipiravir from day 1 and those who received it from day 6. There was also no significant difference in the time to defervescence among patients who had fever on day 1 using the prespecified adjusted Cox proportional hazard method, but the *P* value computed by the generalized Wilcoxon test indicated statistical significance (Fig. 3). The body temperature, adjusted for day 1, was consistently lower between days 2 and 5 in the early treatment group than in the late treatment group until they converged on day 6, and the difference on day 2 was statistically significant. This is in line with the observations that the difference in the viral load decrease was largest between day 1 and day 2 and that the 50% logarithmic reduction in viral load by day 6 was numerically greater in the early treatment group, suggesting the presence of modest antiviral activity in this setting. It has been suggested that antiviral therapy may be more efficacious if given early in the course of COVID-19 than that in the terminal stage of illness (7). Favipiravir, which is active *in vitro* against SARS-CoV-2 and can be administered orally, is an agent which may fit this paradigm. The fact that none of the patients in this study had progressive disease or death is reassuring. It is not clear, however, why viral clearance in the nasopharynx by RT-PCR was less robust in this study than in earlier studies that used formulations of favipiravir which are expected to be equivalent to Avigan (5, 6). From a mechanistic standpoint, favipiravir is expected to limit viral replication but may not necessarily expedite clearance of SARS-CoV-2. The prespecified subgroup analysis indicated that a significantly higher rate of viral clearance by the sixth day occurred among those whose age was ≥65 and had received favipiravir. However, there was also a significant imbalance between the baseline viral loads; thus, the finding should be interpreted with caution.

The viral loads and viral clearance in RT-PCR were key elements in this study, as they may differ depending on the types of RT-PCR kits and reagents used. In this study, we employed the N2 primer-probe set, which is widely carried out by one-step quantitative RT-PCR (qRT-PCR) targeting SARS-CoV-2 for molecular diagnosis of COVID-19 in Japan (8). The N2 set is one of the more sensitive qRT-PCR assays of the N coding region compared with various primer-probe sets posted through the World Health Organization (8, 9). The N2 set has no cross-reactivity with SARS-CoV or other respiratory viruses. Thus, the N2 set has high specificity and sensitivity for SARS-CoV-2 detection and is reliable for viral RNA quantification (8–10).

The most common adverse event associated with favipiravir is hyperuricemia (4.79% according to the package insert), which is associated with inhibition of OAT1, OAT3, and URAT1 by this agent (11). The incidence was much higher in this trial, at 84.1%, which is likely due to the higher dose used than is approved of for influenza in Japan (1,600 mg twice followed by 600 mg twice a day [b.i.d.]). There was no apparent incremental increase in the serum uric acid level after 5 to 10 days of administration; thus, the effect appeared to be dose dependent rather than cumulative. Hyperuricemia was transient and resolved in most patients who stayed in hospital or returned for blood draw ≥5 days from the last dose of favipiravir.

Several limitations are notable in our study. First, the sample size of this study was based on an earlier report of a nonrandomized study from China, which reported highly significant viral clearance rates in the first 5 days of therapy with favipiravir plus interferon alpha compared with lopinavir-ritonavir plus interferon alpha. The effect size in our randomized setting was smaller, which along with an unexpected high frequency of a negative RT-PCR at the time of enrollment likely underpowered the study. Second, the open-label study design may have biased assessment and management outside the trial. Third, the staggered treatment design where all patients eventually received favipiravir, adopted due to the unavailability of placebo at the time of study conception, made it difficult to interpret outcome differences beyond the sixth day. Fourth, the study only recruited asymptomatic to mildly symptomatic COVID-19 patients, and the findings cannot be extrapolated to patients with moderate to severe disease. Enrolling only patients without symptoms or with mild disease also made it difficult to assess symptoms, possibly underestimating potential clinical benefits of favipiravir. Finally, virological endpoints were assessed by RT-PCR only, and it is not known whether early treatment had any impact on replication-competent viruses.

In summary, in this randomized trial of patients with asymptomatic to mildly symptomatic COVID-19, administration of favipiravir did not significantly improve viral clearance in the first 6 days, but there was a trend toward earlier viral clearance with the agent. Favipiravir was associated with numerical reduction in time to defervescence, and a significant improvement in fever was observed the day after starting therapy, compared with findings with no therapy. None of the patients experienced progression of disease or death. While limited by the small sample size, the findings suggest antiviral activity of favipiravir in this patient population. Further studies are required to demonstrate whether this effect translates to prevention of disease progression and mortality.

## MATERIALS AND METHODS

### Study design.

This was an investigator-initiated, individually randomized, open-label trial to assess the efficacy and safety of oral favipiravir in adolescents and adults (aged ≥16 years) admitted to hospital with asymptomatic to mildly symptomatic COVID-19.

The study was centrally approved by the certified review board of Fujita Health University, which served as the coordinating center, and subsequently approved by the director of each participating hospital prior to site initiation. Written informed consent was obtained from all study participants. The trial protocol is available in the supplemental material.

### Patients.

Patients were recruited at 25 hospitals across Japan. The study recruitment period was from 2 March 2020 to 18 May 2020. The follow-up was completed on 14 June 2020.

Inclusion criteria were the following: (i) age of 16 years or older, (ii) inpatient status, (iii) positive RT-PCR for SARS-CoV-2 from a pharyngeal or nasopharyngeal swab specimen collected within 14 days, (iv) Eastern Cooperative Oncology Group (ECOG) performance status of 0 or 1 (12), (v) ability to remain hospitalized for 6 days or longer, (vi) negative pregnancy test (premenopausal female only), and (vii) written consent for participation.

Exclusion criteria were the following: (i) performance status of 2 or greater, (ii) severe hepatic disease, (iii) need for dialysis, (iv) altered mental status, (v) pregnancy, (vi) female patients who did not agree to use effective contraceptive methods, (vii) male patients with female partners who did not agree to the use of effective contraceptive methods, (viii) hereditary xanthinuria, (ix) hypouricemia or history of xanthine urolithiasis, (x) uncontrolled gout or hyperuricemia, (xi) immunosuppressive conditions, and (xii) receipt of systemic antiviral agent against SARS-CoV-2 within 28 days.

### Randomization.

Potential study participants were screened for eligibility within 24 h prior to the study randomization. Patients were randomly assigned via computer-generated random numbering (1:1) to start favipiravir either on day 1 (early treatment group) or on day 6 of study participation (late treatment group). An open-label design was adopted, as placebo was not readily available at the time of study initiation. Treatment was staggered by 5 days between the two groups to allow for a sufficient window to directly compare effects of treatment versus no treatment while keeping the delay within a clinically reasonable period in the late treatment group. The randomization was stratified based on age (≥65 or <65) and days between collection of the SARS-CoV-2-positive specimen and enrollment (<8 days and ≥8 days). Patients and clinicians were not masked to treatment assignment.

### Procedures.

Favipiravir was dosed at 1,800 mg twice orally at least 4 h apart on the first day, followed by 800 mg orally twice a day, for a total of up to 19 doses over 10 days. This regimen achieves plasma concentration of approximately 60 μg/ml and higher in healthy individuals (data on file, Fujifilm Toyama Chemical). If the patients met the discharge criteria (resolution of symptoms and two serial negative results for RT-PCR performed locally) which were sanctioned by the government during the study period, and they had reached at least the sixth day of study participation, they were allowed to discontinue favipiravir, be discharged home, and followed up at the end of the study visit. Use of other medications with antiviral activity was prohibited during the course of study participation.

Nasopharyngeal swabs were collected daily between day 1 and day 6 and then every other day through day 16 if the patients remained in hospital. RT-PCR was conducted at a centralized study laboratory using the protocol that was developed at the National Institute of Infectious Diseases and widely adopted in Japan (10). Briefly, SARS-CoV-2 RNA was extracted from 200 μl of the viral transport medium with a MagMax CORE nucleic acid purification kit by using the King Fisher Flex purification system (Thermo Scientific, Bremen, Germany). A QuantiTect probe RT-PCR kit (Qiagen, Hilden Germany) was used to determine RNA copy numbers in 5 μl of purified viral RNA by the N2 primer-probe set, in which the forward (NIID_2019-nCOV_N_F2; 5′-AAATTTTGGGGACCAGGAAC-3′) and reverse (NIID_2019-nCOV_N_R2; 5′-TGGCAGCTGTGTAGGTCAAC-3′) primers were used to amplify a 119-bp segment of the viral RNA with the dual fluorophore-labeled probe (NIID_2019-nCOV_N_P2; 5′-FAM-ATGTCGCGCATTGGCATGGA-TAMRA-3′). The thermal cycling conditions were as follows: 50°C for 30 min, 95°C for 15 min, and 45 cycles of 95°C for 15 s and 60°C for 1 min. Amplified products were monitored with a LightCycler 480 II (Roche Diagnostics, Basel, Switzerland), and the copy number in each sample was determined based on the standard curve produced by the synthesized control RNA.

### Outcomes.

The primary outcome endpoint was time to SARS-CoV-2 clearance and presence or absence of SARS-CoV-2 clearance by RT-PCR of nasopharyngeal specimens by day 6. For the late treatment group, the specimen was collected prior to initiation of favipiravir on day 6.

Secondary outcome endpoints were as follows: (i) SARS-CoV-2 clearance by day 10, (ii) 50% logarithmic reduction in the SARS-CoV-2 viral load by day 6, (iii) changes in logarithmic SARS-CoV-2 viral load, and (iv) time until SARS-CoV-2 clearance by local RT-PCR. Exploratory outcome endpoints were as follows: (i) duration of fever (≥37.5°C or ≥37.0°C), (ii) change in body temperature, (iii) change in symptoms, assessed by the number of symptoms, including subjective fever, cough, sore throat, headache, myalgia or arthralgia, chills or diaphoresis, fatigue, disturbance of consciousness, shortness of breath, rhinorrhea, chest pain, diarrhea, and nausea or vomiting, (iv) disease progression or death, and (v) death.

### Statistical analysis.

The sample size was calculated upon the assumption that the virus would be undetectable by day 6 in 80% of the subjects in the early treatment group and 50% of the subjects in the late treatment group, based on a prior study (5). With a power of ≥80% and a two-sided significance level of 0.05, the study required 39 patients in each group. Accounting for a 10% dropout rate, 43 subjects were required in each arm.

According to the study protocol that was *a priori* planned before the data analysis, unless otherwise stated, analyses for virological endpoints were performed on the infected ITT population. This was defined as all subjects who enrolled into the study except those for whom there was no efficacy data after randomization occurred and those whose first RT-PCR result on day 1 was already negative. Analyses for clinical endpoints were performed on the ITT population, defined as infected ITT without exclusion of those whose first RT-PCR result on day 1 was already negative.

Statistical analysis was performed on randomly assigned treatment groups. Continuous variables were summarized by presenting the median and IQR. Categorical variables were summarized by presenting the frequency and proportion of patients in each category. Time-to-event data were analyzed with the Cox proportional hazard regression model. Kaplan-Meier survival curves were computed as the cumulative proportion of viral clearance where the statistical significance was tested with the generalized Wilcoxon test.

Fifty-percent logarithmic viral load reduction was considered present if the following logarithmic viral load reduction (percent) was 50 or greater, which was defined as [(logarithmic viral load on day 1 − logarithmic viral load on day 6)/(logarithmic viral load on day 1 – logarithmic limit of detection)] × 100 where the limit of detection was set to 120 copies/ml. Logistic regression was used to compute the odds ratio of the 50% logarithmic viral load reduction.

To assess changes in viral load, body temperature, and symptoms, intergroup comparisons were conducted with the repeated measures regression model using the mixed-effect (MIXED) model. In order to assess change from the baseline in the model with the logarithmic viral load as an outcome variable, the baseline logarithmic viral load was further adjusted in the model. Similarly, Cox proportional hazard regression was used to compare time to defervescence, which was defined as the day body temperature became less than 37.5°C among patients whose body temperature was greater than 37.5°C at baseline. To compare average change in body temperature from the baseline, the mixed-effect regression model was used where the body temperature at each time point was the outcome variable, and the regression model further adjusted for the baseline body temperature. Change in symptom score was analyzed using the generalized estimation equation (GEE) regression. All regression analyses mentioned above were adjusted for age and days between collection of the SARS-CoV-2-positive specimen and enrollment.

As *a priori*-decided exploratory subgroup analyses, subgroups were generated based on the initial SARS-CoV-2 viral load, presence or absence of symptoms, age of ≥65 and <65, and days from the first positive RT-PCR result prior to study enrollment to examine if efficacy was observed in these subgroups. Cox proportional hazard regression models were used in the subgroup analysis without covariate adjustment due to small sample size. Statistical analyses were performed with SAS software, version 9.4 (SAS Institute, Cary, NC).

## Supplementary Material

Supplemental file 1
