# Supplementary material for: A Prospective, Randomized, Open-Label Trial of Early versus Late Favipiravir Therapy in Hospitalized Patients with COVID-19
Source: Antimicrob Agents Chemother. 2020 Nov 17;64(12):e01897-20. doi: 10.1128/AAC.01897-20 (PMC7674035; doi:10.1128/AAC.01897-20)
Supplement: Supplemental file 1 [file AAC.01897-20-s0001.pdf]

Supplemental Table 1. Baseline demographics and clinical characteristics of COVID-19 patients in the Infected ITT population.

	**Early treatment (n = 36)**	**Late treatment (n = 33)**	**Infected ITT population (n=69)**
**Demographic and clinical characteristics**			
Age, median (ICR), y	48.0 [31.5, 64.5]	50.0 [40.0, 59.0]	49.0 [38.0, 62.0]
Sex, No. (%)			
Male	17 (47.2%)	24 (72.7%)	41 (59.4%)
Female	19 (52.8%)	9 (27.3%)	28 (40.6%)
Body mass index, median (ICR)	22.0 [20.4, 24.5]	23.4 [21.9, 25.8]	22.8 [20.8, 24.9]
Coexisting diseases, No. (%)	12 (33.3%)	16 (48.5%)	28 (41%)
Symptomatic, No. (%)	26 (72%)	25 (76%)	51 (74%)
Subjective fever, No. (%)	13 (36.1%)	16 (48.5%)	29 (42%)
Cough, No. (%)	17 (47.2%)	10 (30.3%)	27 (39%)
Dyspnea, No. (%)	2 (5.6%)	4 (12.1%)	6 (9%)
Myalgia or arthralgia, No. (%)	3 (8.3%)	3 (9.1%)	6 (9%)
Need for supplemental oxygen at randomization, No (%)	1 (2.8%)	2 (6.1%)	3 (4.3%)
Time between fever onset and randomization, median (IQR), d*	7.0 [4.0, 9.0]	7.5 [5.00, 10.0]	7.0 [5.0, 9.0]
Time between positive PCR and randomization, median (IQR), d	4.0 [3.0, 4.5]	4.0 [2.0, 5.0]	4.0 [2.0, 5.0]
**Laboratory values**			
SpO_2, median, %	96.0 [95.0, 97.0]	96.0 [95.0, 97.0]	96.0 [95.0, 97.0]
Body temperature, median (IQR), °C	37.2 [36.8, 38.0]	37.2 [36.8, 38.0]	37.2 [36.8, 38.0]
Systolic blood pressure, median (IQR), mmHg	122.0 [116.0, 136.0]	120.0 [112.0, 127.0]	120.0 [114.0, 129.0]
Heart rate, median (IQR), /min	77.5 [73.0, 84.5]	75.0 [66.0, 81.0]	77.0 [68.0, 84.0]
Respiratory rate, median (IQR), /min	16.5 [16.0, 22.0]	18.0 [16.0, 19.0]	18.0 [16.0, 20.0]
White blood cell count, median (IQR), cells/uL	4.3 [3.3, 5.2]	4.6 [3.9, 6.2]	4.4 [3.6, 5.8]
Platelet count, median (IQR), $x10^3$/uL	182.5 [135.0, 230.0]	198.0 [164.0, 241.0]	190.0 [155.0, 238.0]
CRP, median (IQR), mg/L	1.4 [0.2, 4.9]	0.8 [0.2, 2.4]	1.1 [0.2, 3.9]
ALT, median (IQR), U/L	17.5 [11.5, 29.0]	18.0 [16.0, 33.0]	18.0 [14.0, 29.0]
AST, median (IQR), U/L	22.5 [18.0, 29.0]	23.0 [19.0, 31.0]	23.0 [18.0, 30.0]
Urea nitrogen, median (IQR), mg/dL	12.4 [9.9, 14.5]	12.2 [10.8, 15.6]	12.2 [10.0, 14.7]
Serum creatinine, median (IQR), mg/dL	0.7 [0.6, 0.9]	0.9 [0.7, 1.0]	0.8 [0.6, 0.9]
Uric acid, median (IQR), mg/dL	4.1 [3.0, 5.1]	4.8 [4.0, 5.8]	4.5 [3.4, 5.3]
Viral load (IQR), copies/mL	$10^{4.7}$ [$10^{3.4}$, $10^{6.5}$]	$10^{5.4}$[$10^{4.5}$, $10^{6.7}$]	$10^{5.3}$[$10^{3.8}$, $10^{6.5}$]

*n=27 in the early treatment group, n=22 in the late treatment group.

Supplemental Figure legends

Figure S1. Average change in viral load from baseline among the infected intention-to-treat population. P values were computed with the mixed effect regression model adjusted for log-transformed baseline viral load, age and days between collection of the SARS-CoV-2-positive specimen and enrollment.

Figure S2. Average change in body temperature from baseline among the intention-to-treat population. P values were computed with the mixed effect regression model adjusted for baseline body temperature, age and days between collection of the SARS-CoV-2-positive specimen and enrollment.

Figure S3. Average change in 4 major symptoms from baseline among the intention-to-treat population. P values were computed with the generalized estimating equation (GEE) regression model adjusted for baseline score, age and days between collection of the SARS-CoV-2-positive specimen and enrollment.

Figure S4. Average change in 13 major symptoms from baseline among the intention-to-treat population. P values were computed with the generalized estimating equation (GEE) regression model adjusted for baseline score, age and days between collection of the SARS-CoV-2-positive specimen and enrollment.

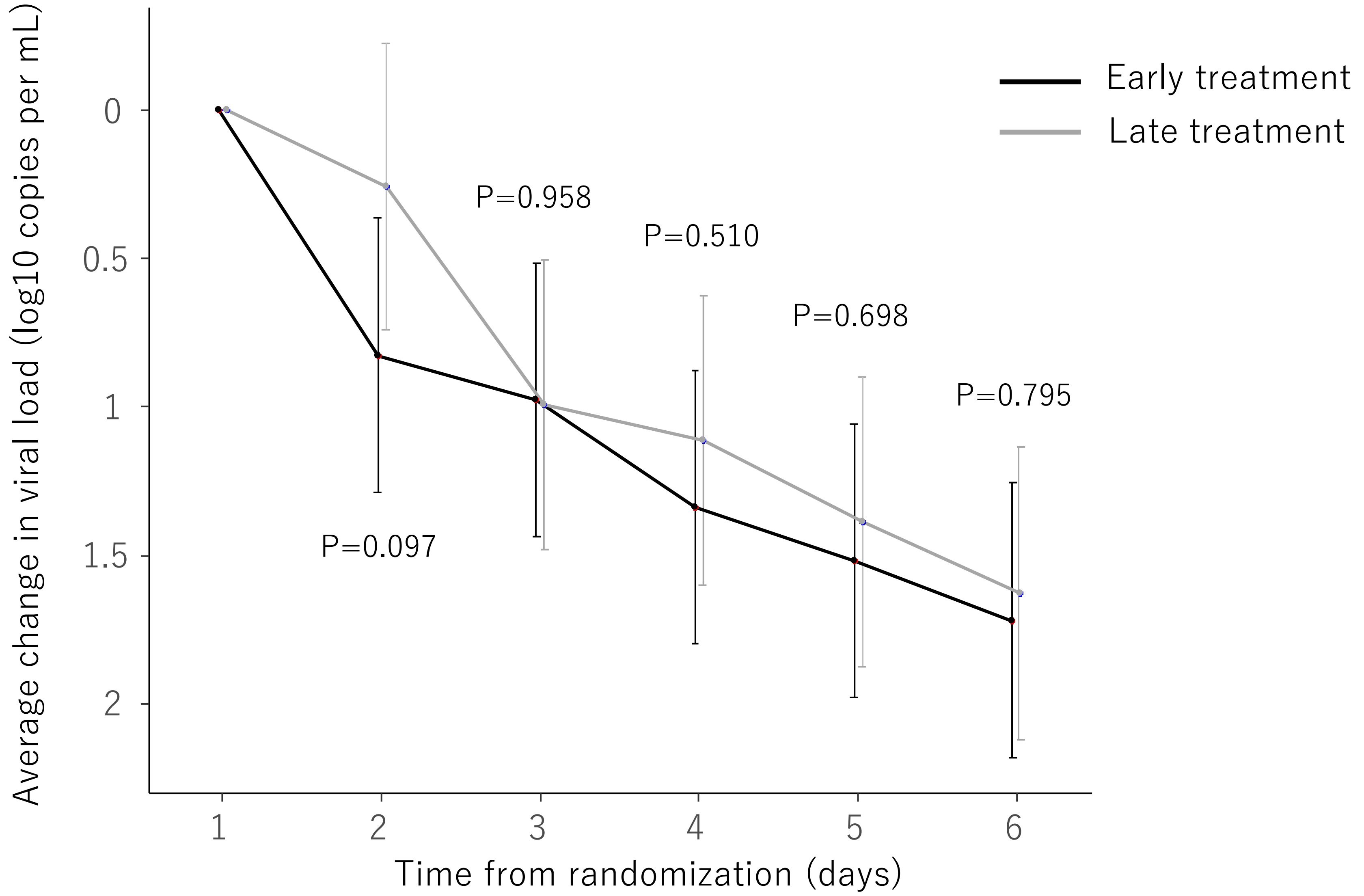

Figure S1. Average change in viral load from baseline among the infected intention-to-treat population. P values were computed with the mixed effect regression model adjusted for log-transformed baseline viral load, age and days between collection of the SARS-CoV-2-positive specimen and enrollment.

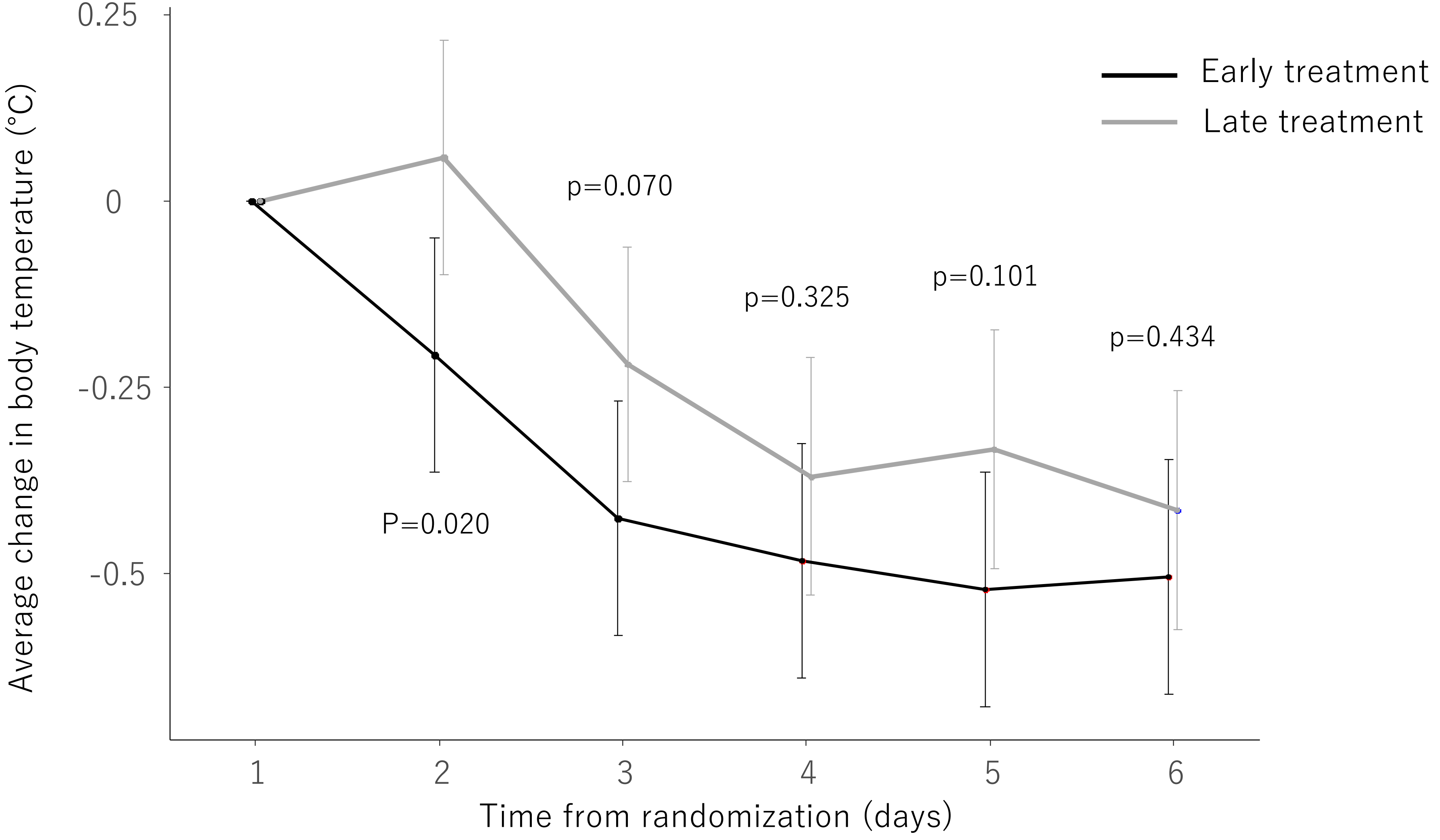

Figure S2. Average change in body temperature from baseline among the intention-to-treat population. P values were computed with the mixed effect regression model adjusted for baseline body temperature, age and days between collection of the SARS-CoV-2-positive specimen and enrollment.

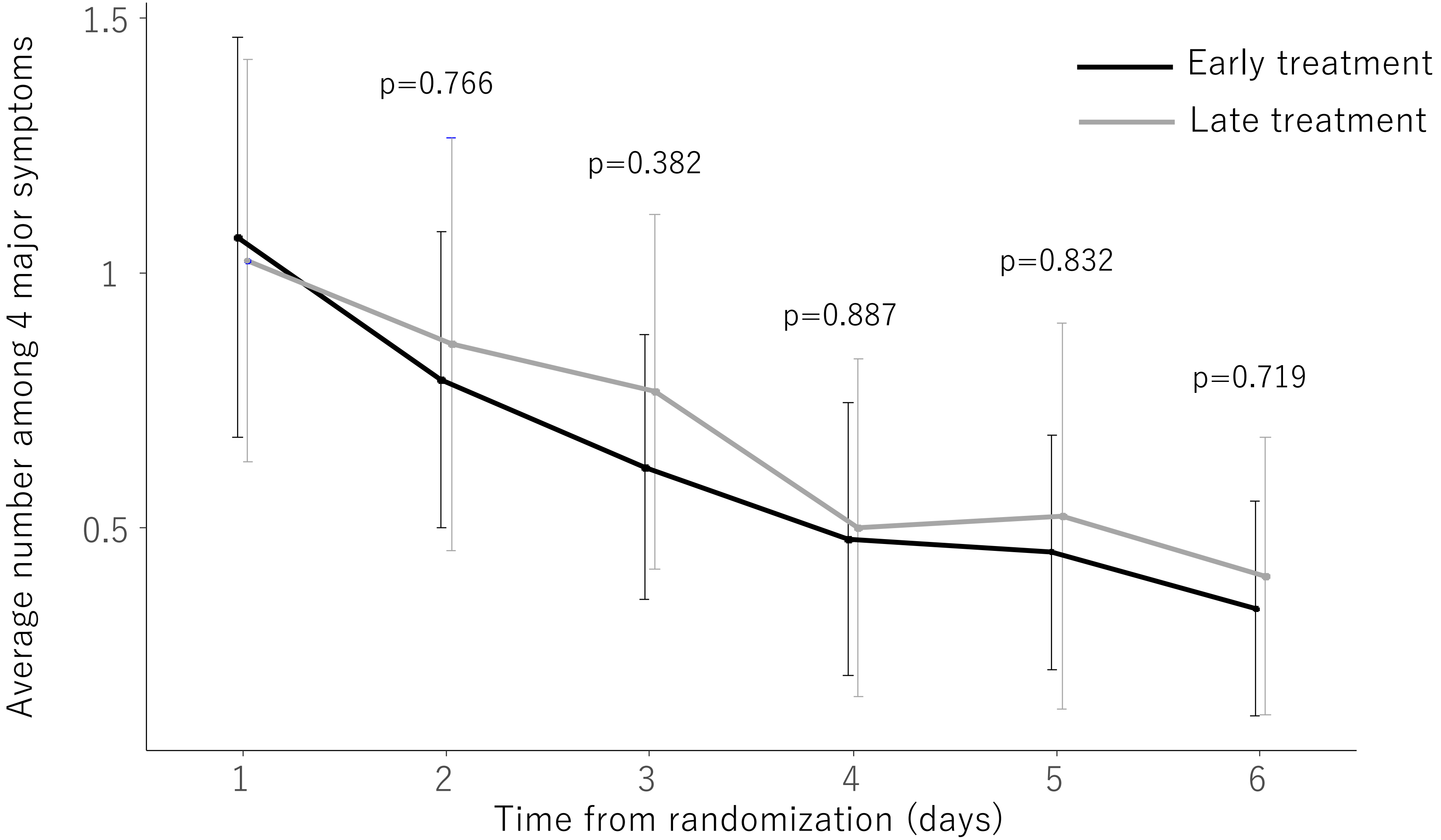

Early treatment	43	43	42	44	42	44
Late treatment	42	43	43	42	42	42

Figure S3. Average change in 4 major symptoms from baseline among the intention-to-treat population. P values were computed with the generalized estimating equation (GEE) regression model adjusted for baseline score, age and days between collection of the SARS-CoV-2-positive specimen and enrollment.

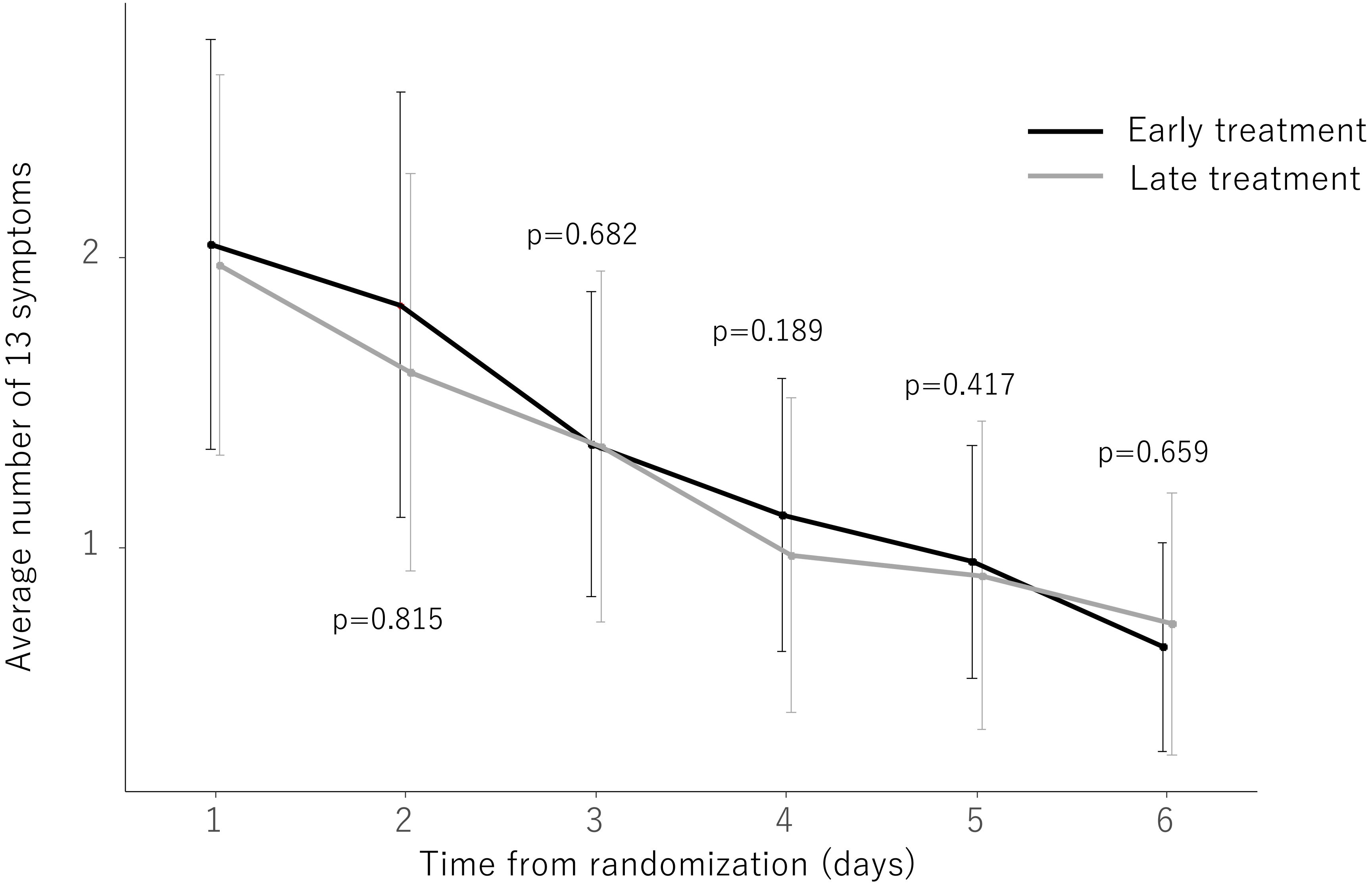

Early treatment	43	43	42	44	42	44
Late treatment	42	43	43	42	42	42

Figure S4. Average change in 13 major symptoms from baseline among the intention-to-treat population. P values were computed with the generalized estimating equation (GEE) regression model adjusted for baseline score, age and days between collection of the SARS-CoV-2-positive specimen and enrollment.

Multicenter, open-label, randomized trial of favipiravir in asymptomatic and mildly symptomatic patients infected with SARS-CoV-2 to evaluate viral load reduction

Study Protocol

Fujita Health University
National Institute of Infectious Diseases

Document: COVID19JP01
Version: 3.00
Date: May 19, 2020

Index

1. Rationale for the study
    a. Background
2. Purpose of the study
3. Ethical considerations
    a. Ethics
    b. Patient consent
    c. Protection of privacy
4. Subjects
    a. Subjects
    b. Inclusion criteria
    c. Exclusion criteria
    d. Duration of study participation
5. Endpoints
    a. Efficacy endpoints
    b. Safety endpoints
6. Study drug
    a. Study drug
    b. Pharmacology, pharmacokinetics, clinical data and safety information
7. Study design
    a. Study design
        i. Treatment allocation
    b. Dosing, dosage and treatment duration
    c. Management of study drug
    d. Registration of subjects
    e. Concomitant medications and therapies
8. Data collection and timeline
    a. Observation items
    b. Schedule
        i. Subject background
        ii. Pregnancy test
        iii. Notification to other entities
        iv. Clinical findings
        v. Laboratory tests
        vi. RT-PCR testing
        vii. Vital signs
        viii. Study drug administration
        ix. Duration of hospitalization
        x. Assessment of pneumonia
    c. Risk management for the subjects
        i. Information on adverse events of favipiravir
        ii. Potential direct benefits to the subjects
        iii. Risk mitigation
9. Safety evaluation
    a. Adverse events
10. Criteria for evaluation
    a. Adverse events
    b. Serious adverse events
        i. Reporting to the Certified Review Board
        ii. Reporting to the Minister of Health, Labour and Welfare
        iii. Reporting to the provider of study drug

    c. Reporting of pregnancy
    d. Provision of care upon completion of study participation
11. Number of subjects
12. Study duration
13. Criteria for study discontinuation
14. Study discontinuation
    a. Study completion
    b. Study discontinuation
15. Monitoring, auditing and data management
    a. Monitoring and auditing
    b. Access to source data
    c. Data management
    d. Source data
16. Compliance to the study protocol
    a. Compliance to the study protocol
    b. Deviation from the study protocol
17. Statistical analysis
    a. Analysis sets
    b. Efficacy analysis
        i. Primary efficacy endpoint
        ii. Secondary efficacy endpoints
        iii. Exploratory efficacy endpoints
    c. Safety analysis
    d. Others
18. Compensation
19. Secondary use of study data
20. Management and disposal of study data, specimens
21. Registration of study plan and announcement of study data
22. Funding and conflicts of interest
23. Costs to subjects
24. Management of protected health information
25. Reporting to participating institutions and Minister of Health, Labour and Welfare
26. Consultation from subjects and their legal representatives
27. Study organization
28. Contracting
29. References

1. Rationale for the study

Favipiravir is an antiviral agent developed by Toyama Chemical Co. Ltd. (now FUJIFILM Toyama Chemical Co., Ltd.). It was approved for clinical use by the Ministry of Health, Labour and Welfare in March 2014 for "new or emerging influenza infection for which other anti-influenza drugs are ineffective or not sufficiently effective". Its mechanism of action is selective inhibition of viral RNA polymerase in vivo by its triphosphorylated derivative (T-705RTP), which translates to broad spectrum inhibition of RNA viruses beyond the influenza virus. Its in vitro or in vivo activity against Filoviridae, Arenaviridae and Bunyaviridae has been reported in the literature.

1-1. Background

The new coronavirus infection (COVID-19), which started in the City of Wuhan, Hubei Province in People's Republic of China has evolved into a global pandemic in the subsequent four months, causing over four hundred thousand infections and twenty thousand deaths. The new coronavirus (SARS-CoV-2) is believed to have originated from animals but can cause human-to-human infections resulting in pneumonia and sometimes death. Supportive therapy limits s the mainstream approach of clinical care and there is an urgent need to develop prophylactic and therapeutic modalities.

2. Purpose of the study

The purpose of this study is to administer favipiravir for 10 days to patients who have confirmed SARS-CoV-2 infection which is asymptomatic or mild and evaluate its efficacy and safety.

The primary endpoint of this study is the proportion of subjects with a negative SARS-CoV-2 PCR result from the nasopharyngeal swab specimen by Day 6. The secondary endpoints of this study are the proportion of subjects with a negative SARS-CoV-2 PCR result in the nasopharyngeal swab specimen by Day 10 the proportion of subjects with 50% reduction in the SARS-CoV-2 viral load in the nasopharyngeal swab specimen on a logarithmic scale, changes over time in the SARS-CoV-2 viral load in the nasopharyngeal swab specimen, and the time until the first of two serial negative SARS-CoV-2 PCR results as determined by clinically indicated PCR tests. The exploratory endpoints of this study are duration of fever, changes over time in body temperature, changes over time in symptoms attributable to COVID-19, the proportion of subjects who progress to severe disease or death, and the proportion of subjects with death.

3. Ethical considerations

3-1. Ethics

All individuals and organizations involved in the study will conduct it in compliance with the Helsinki Declaration and the Clinical Trials Act (Act No. 16 of April 14, 2017).

3-2. Patient consent

(1) Adults

Informed consent for participation in the study will be obtained from the patient in writing under free will. The study will be explained to the patient with respect to the level of understanding.

(2) Minors age 16 or older

Informed consent for participation in the study will be obtained from the patient in writing under free will. In addition, informed consent with be obtained in writing from the patient's legal representative. The study will be explained to the patient and legal representative with respect to the level of understanding.

A person in parental authority or an adult guardian who can represent the will and interest of the patient is eligible to serve as the legal representative.

(3) Re-consenting

Re-consent is obtained in writing from the subjects by the principal investigator or site investigators to affirm the subjects' will for continued participation when study plans are changed or new information

about the study becomes available that, in the view of the principal investigator, may affect the subjects' will to continue on the study.

Re-consenting is not required if the principal investigator determines that these items would not affect the subjects' will for continued participation.

(4) Loss of consenting capacity by the subject during study participation

If a subject is deemed to be no longer capable of consenting to continued participation in the study due to progression of disease or other causes, a legal representative meeting the below three criteria may re-consent or withdraw consent on behalf of the subject.

- Parent, spouse, child, sibling, person in parental authority, adult guardian or equivalent
- Represents the maximum benefit and welfare of the subject
- Adult age 20 years or older with consenting capacity

Rationale: The study enrolls asymptomatic or mild COVID-19 patients. However, sudden deterioration in clinical conditions may occur, necessitating sedation and/or mechanical ventilation.

3-3. Protection of privacy

All individuals and organizations involved in the conduct of the study must make all efforts to protect the privacy of the subjects in accordance with the Act on the Protection of Personal Information (Act No. 57 of May 30, 2003), including non-use of information that may identify the subjects, such as their names and addresses.

4. Subjects

4-1. Subjects

Patients who had a positive RT-PCR test for SARS-CoV-2 from a pharyngeal or nasopharyngeal swab specimen, meet all the inclusion criteria, and do not meet any of the exclusion criteria.

4-2. Inclusion criteria

(1) Age 16 or older on the day of enrollment
(2) Sex: any
(3) Patient care setting: inpatient only
(4) The patient meets all three criteria below
  a. Had a positive RT-PCR test for SARS-CoV-2 from a pharyngeal or nasopharyngeal swab specimen collected within 14 days prior to enrollment
  b. Has a performance status of 0 or 1
  c. Able to remain hospitalized for 6 days or longer
(5) The patient has a negative pregnancy test (pre-menopausal female only)
(6) The patient has provided written consent for participation (written consent from both the patient and legal representative is required for minors age 16 or older)

Rationale:

(1) Effective medical treatment has not been established for SARS-CoV-2 infection, and minors may also develop infection in the setting of the pandemic.

(4) The criteria take into consideration the study aim, which is evaluation of viral load reduction among asymptomatic and mild patients.

(5) Pregnancy test is required since the safety of the drug for fetuses and pregnant women has not been established.

(6) The criterion is based on the Clinical Trials Act (Act No. 16 of April 14, 2017).

4-3. Exclusion criteria

The below exclusion criteria should be confirmed at the time of enrollment.

(1) Performance status of 2 or greater
(2) Severe hepatic disease corresponding to Grade C by the Child-Pugh classification
(3) Need for dialysis for impaired renal function

(4) Altered mental status such as disorientation
(5) Pregnant, or possibility of pregnancy
(6) Female patients who do not agree to use effective contraceptive methods such as oral contraceptive use, use of mechanical contraceptive methods including intrauterine device, pessary or condom, or combinations thereof, from the start of favipiravir administration to 90 days after the end of favipiravir administration
(7) Male patients with female partners who do not agree to the use of contraceptive methods as described in (6)
(8) Male patients who do not agree to the use of condoms from the start of favipiravir administration to 90 days after the end of favipiravir administration
(9) Patients with hereditary xanthinuria
(10) Patients with hypouricemia (≤1 mg/dL) or history of xanthine urolithiasis
(11) Patients with uncontrolled gout or hyperuricemia
(12) Patients with immunosuppressive conditions such as HIV positivity
(13) Patients who received systemic antiviral agent against SARS-CoV-2 within 28 days prior to enrollment
(14) Patients who in the view of the principal investigator or site investigators are unfit to undergo the study

Rationale:
(1) Moderate to severe disease is excluded from the study.
(2) The exposure (AUC) of favipiravir may increase approximately 6-fold in patients with severe liver disease.
(3) Plasma concentration of favipiravir is not maintained in patients undergoing dialysis.
(4) Altered mental status may increase risk of aspiration pneumonia due to the large number of tablets that are administered.
(6)(7) Favipiravir is contraindicated in pregnancy since it has caused fetal deaths and teratogenicity in animals.
(8) Favipiravir has been detected in semen.
(9)(10) Xanthine oxidase deficiency may increase plasma concentration of favipiravir.
(11) Favipiravir has been associated with hyperuricemia.
(12) Immunosuppressive state may affect evaluation of efficacy and safety of favipiravir.
(13) Prior antiviral therapy may affect evaluation of efficacy of favipiravir.

4-4. Duration of study participation

The duration of study participation is 28 days. At least the first 6 days of the study is conducted in hospital. Favipiravir is only administered in hospital.

5. Endpoints

5-1. Efficacy endpoints

Please see Section 17-2 for efficacy endpoints.

5-2. Safety endpoints

Please see Section 17-3 for safety endpoints.

6. Study drug

6-1. Study drug

The study drug favipiravir will be donated by FUJIFILM Toyama Chemical Co., Ltd.
(1) Brand name: Avigan 200 mg Tablet
(2) Generic name: favipiravir (JAN)
(3) Chemical name: 6-Fluoro-3-hydroxypyrazine-2-carboxamide
(4) Chemical structure:

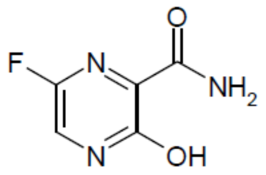

(5) Molecular formula: $C_5H_4FN_3O_2$
(6) Molecular weight: 157.10
(7) Dosage form: film-coated tablet, light yellow
(8) Content: 200 mg of favipiravir per tablet
(9) Manufacturer: FUJIFILM Toyama Chemical Co., Ltd.
(10) Storage: room temperature, designated as a powerful drug

6-2. Pharmacology, pharmacokinetics, clinical data and safety information

Information on the pharmacology, pharmacokinetics, clinical data and safety of favipiravir can be found in the package insert. A summary of the domestic high-dose 22-day administration study (JP120 Study), which is not included in the package insert, is provided below:

Favipiravir was administered in a randomized, double-blind, placebo-controlled study to healthy adult men age 20 to 39 at 1,800 mg twice on Day 1 followed by 800 mg twice a day for a total of 22 days to evaluate safety, tolerability and pharmacokinetics.

The peak plasma concentrations of favipiravir ranged between 87 and 104 ug/mL after Day 5, and the Cmin ranged between 56 and 75 ug/mL after the second dose on Day 1. Favipiravir was mostly undetectable in the plasma on Day 29 (7 days after the last dose).

Cmin of favipiravir above 60 ug/mL would be required when considering the IC_{99}, EC_{50} or EC_{90} values of Filoviridae including the Ebola virus, Arenaviridae and Bunyaviridae, and the protein binding of approximately 50% in humans. The Cmin of 56 to 75 ug/mL demonstrated by the 1800/800 mg BID dosing in this study suggests potential efficacy against these viruses.

Adverse events whose causal relationship with favipiravir administration could not be ruled out were observed in all 8 subjects for a total of 14 events (increase in uric acid in 8 subjects, increase in ALT in 2 subjects, increase in AST in 2 subjects, upper abdominal pain in 1 subject, and rash in 1 subject). All these events were mild and resolved with discontinuation of favipiravir. No serious adverse events were observed.

7. Study design

7-1. Study design

Multicenter, open-label, randomized study

The subjects are randomly assigned to the favipiravir early treatment group and the delayed treatment group at a 1:1 ratio.

7-1-1. Treatment allocation

Allocation of the subjects to the treatment arms will be managed centrally using random allocation by the stratified permuted block method. The allocation stratification variables will include 1) site, 2) age (65 or greater and less than 65), 3) days between collection of the SARS-CoV-2-positive specimen and enrollment (less than 8 days and 8 days or greater where the same day is defined as one day). The procedures for randomization will be managed by the allocation manager according to the allocation manual that will not be made public.

7-2. Dosing, dosage and treatment duration

Favipiravir will be administered orally at 1,800 mg twice on Day 1 and 800 mg twice a day from Day 2 in the early treatment group for up to a total of 10 days (only the morning dose is given on Day 10). In the

delayed treatment group, favipiravir will be administered orally starting on Day 6 up to a total of 10 days. The subjects should remain hospitalized until the day following the last day of favipiravir administration. However, if a subject has reached Day 5 with all symptoms resolved and meets the discharge criteria set forth by the MHLW, favipiravir may be stopped at that point and the subject can be discharged the following day after clinical observation and laboratory testing has been performed. Favipiravir cannot be administered after discharge.

On the first day of treatment, the two doses are administered at least 4 hours apart.

If oral administration is not possible due to the subject's clinical status, the drug may be suspended in water at 55°C (simple suspension method). The suspension will be administered through a nasogastric tube whose location has been verified, followed by flushing with 5 ml of water.

In the delayed treatment group, if the illness progresses by Day 5 and the site investigator feels that early administration of favipiravir is clinically desirable from an ethical perspective, early administration can be considered in consultation with the principal investigator. In such an instance, the subject will remain on study and otherwise continue with the schedule of events.

Rationale: The dose was selected based on the maximum safe dose which can be expected to be safely administered based on dose-escalation studies and inhibit SARS-CoV-2 in vivo.

7-3. Management of study drug

The principal investigator and the study pharmacist will record the inventory status of the study drug. They will investigate, reconcile and document any discrepancies.

The study drug for this study will be maintained and managed separately from the study drug for any other studies or compassionate programs.

7-4. Registration of subjects

The principal investigator or a site investigator will start administration of favipiravir after assigning an identification code to the subject.

The principal investigator will document the name, consent date, subject identification code, enrollment date in the order of the subject identification codes and generate their list.

7-5. Concomitant medications and therapies

(1) Concomitant medications

The principal investigator or a site investigator will record the names, routes, daily doses, durations and indications of concomitant medications from the day of enrollment to the last day of favipiravir administration in the case report form.

If an adverse event occurs that requires follow-up beyond the end of favipiravir administration or discontinuation, concomitant medications administered to address the adverse event of interest should also be investigated through the last day of study participation.

(2) Prohibited medications

The below medications are prohibited during the study participation since they may interfere with the evaluation of the efficacy of favipiravir.

1) Interferon-alpha
2) Remdesivir
3) Chloroquine and hydroxychloroquine
4) Lopinavir-ritonavir
5) Ciclesonide
6) Nafamostat
7) Camostat
8) Tocilizumab
9) Any other medications for which antiviral activity against SARS-CoV-2 has been suggested

However, if subjects in the delayed treatment group experience progression of disease by Day 5, the site investigator may consult the principal investigator and provide necessary clinical care including medications.

(3) Medications requiring caution

The below medications should be administered with caution as drug-drug interaction with favipiravir may occur. Favipiraivr is not metabolized by cytochrome P-450 (CYP); it is metabolized by aldehyde oxidase and partly by xanthine oxidase. Favipiravir inhibits aldehyde oxidase and CYP2C8, but the induction of CYP has not been documented.

1) Pyrazinamide
2) Repaglinide
3) Theophylline
4) Famciclovir
5) Sulindac

Rationale:

1) Pyrazinamide may additively increase reabsorption of uric acid and cause an increase in serum uric acid levels.
2) Serum concentrations of repaglinide may increase due to CYP2C8 inhibition by favipiravir, leading to a higher likelihood of adverse events by repaglinide.
3) Theophylline may increase serum concentration of favipiravir through xanthine oxidase activity, resulting in a higher likelihood of adverse events by favipiraivir.
4) Activity of famciclovir may be diminished by inhibition of aldehyde oxidase by favipiravir.
5) Activity of sulindac may be diminished by inhibition of aldehyde oxidase by favipiravir.

(4) Concomitant therapies

The site investigator will record all concomitant therapies that were administered between enrollment and the last day of favipiravir administration, including types of therapies, dates administered and purposes, to the case report form. Concomitant therapies administered for the purpose of addressing adverse events will be recorded through the end of study participation if such events occurr and evaluation is required beyond the last day of favipiravir administration.

8. Data collection and timeline

8-1. Observation items

Clinical findings, laboratory values, viral testing results (RT-PCR), vital signs (SpO_2, body temperature, blood pressure, pulse, respiratory rate), and adverse events will be collected from the day of enrollment to the last day of favipiravir administration. Data will also be collected after the last day of favipiravir administration if deemed necessary by the principal investigator or site investigators. Viral load will be quantified by RT-PCR using nasopharyngeal swabs.

Rationale: The items and the tests were selected based on safety data from previous studies.

8-2. Schedule

The duration of study participation is 28 days.

Table 1. Study schedule

Time / Item		Pre-study period	Study period (Day 1 is the first day of favipiravir administration or equivalent)																		
		Day -14~ Day 1	Day 1 (pre-dose)	Day 1 (post-dose)	Day 2	Day 3	Day 4	Day 5	Day 6	Day 7	Day 8	Day 9	Day 10	Day 11	Day 12	Day 13	Day 14	Day 15	Day16	Early discharge [i]	Day 28 (±2)	Study discontinuation
Informed consent		●																				
Enrollment			●																			
Patient background			●																			
Pregnancy testing [a]		●																			●[e]	●
Study medication [j]	AM			▼	▼	▼	▼	▼	■[b]	■[b]	■[b]	■[b]	■[b]	▲[b]	▲[b]	▲[b]	▲[b]	▲[b]				
	PM			▼	▼	▼	▼	▼	■[b]	■[b]	■[b]	■[b]	▲[b]	▲[b]	▲[b]	▲[b]	▲[b]					
Clinical findings [k]		◎[d]	●		●	●	●	●	●	◆	◆	◆	◆	◆	◆	◆	◆	◆		●	●[e]	●
Laboratory tests [f, k]		◎[d]	●[h]						●[c]					◆[c]					◆[c]	●	○	○
Nasopharyngeal swab for RT-PCR [g, k]		●[d]	●		●	●	●	●	●		◆		◆		◆		◆		◆	○		
Vital signs [k]		◎[d]	●		●	●	●	●	●	◆	◆	◆	◆	◆	◆	◆	◆	◆		●	○	○
Mandatory hospitalization			←	—	—	—	—	—	→													
Hospitalization [b]			←	—	—	—	—	—	—	—	—	—	—	—	—	—	—	—	→			
Adverse events [l]			←	—	—	—	—	—	—	—	—	—	—	—	—	—	—	—	—	→		

●: Mandatory ▼■: Early treatment group ■▲: Delayed treatment group ◎: Collect if available ◆: Mandatory if in hospital ○: As needed

a: Only required for pre-menopausal women
b: Subjects should remain inpatient until safety laboratory tests are obtained the day following the last day of favipiravir. However, only if the subject has reached Day 5, has clinically improved and meets the discharge criteria sanctioned by the MHLW. After Day 7, the subject may be discharged the day after the last administration of favipiravir. Evaluation after discharge will be performed as needed.
c: Can be performed the following day except for Day 6 in the delayed treatment group. Laboratory tests on Day 6 must be performed prior to favipiravir administration on Day 6 or on Day 5.
d: Must be performed prior to the first dose of favipiravir; can be substituted with information from the referring institution.
e: Can be performed at home or over the phone to collect clinical findings and COVID-19-related symptoms.
f: Collect extra specimens (14-20 mL of blood to yield 8 mL of serum, which should be aliquoted into four cryotubes) for research purposes at each blood draw for laboratory tests, except for Day 28.
g: For eligibility assessment, either a nasopharyngeal or oropharyngeal swab specimen can be used. Swabs after Day 1 should be nasopharyngeal.
h: Data from the day before are acceptable.
i: Should be performed the day following the last dose of favipiravir if early discharge is planned. If discharge is planned before Day 6, the nasopharyngeal swab specimen for that day should be collected before discharge.
j: Only allowed in hospital; favipiravir should be discontinued by the day before discharge.
k: Only required in hospital, except for Day 28.
l: Adverse events should be collected until the day following the last dose of favipiravir.

8-2-1. Subject background

After obtaining informed consent, the principal investigator or site investigator will obtain the below information and record it in the case report form.

(1) Subject ID
(2) Day of consent
(3) Sex
(4) Race and nationality
(5) Date of birth
(6) Weight
(7) Height
(8) Comorbidities
(9) Underlying illnesses that may affect the disease being studied
(10) History of allergies
(11) Days of onset of fever and/or symptoms if present

8-2-2. Pregnancy test

For pre-menopausal women, a pregnancy test must be performed on Day 1 prior to first dose of favipiravir and on Day 28 or upon study discontinuation, with the results recorded in the case report form.

8-2-3. Notification to other entities

The principal investigator or site investigator will identify other services or institutions where the subject sought medical care prior to enrollment and before the first dose of favipiravir, and inform them of the subject's participation to the study as appropriate.

8-2-4. Clinical findings

The principal investigator or site investigator will record daily signs and symptoms (fever, cough, sore throat, headache, myalgia or arthralgia, chills or diaphoresis, fatigue, shortness of breath, rhinorrhea, chest pain, diarrhea, nausea or vomiting, dehydration, dysgeusia or dysosmia) from Day 1 to Day 15 in the case report form. Data prior to enrollment will also be recorded if available. Clinical findings on Day 28 can be obtained by the principal investigator or site investigator by phone. In addition, any other symptoms related to COVID-19 or symptoms that develop after discharge will also be recorded in the case report form.

8-2-5. Laboratory tests

The principal investigator or site investigator will order the laboratory tests listed on Table 2 for Days 1, 6, 11, 16 or at study discontinuation while in hospital, and record the results in the case report form. Any data prior to enrollment should also be captured in the case report form.

Table 2. Laboratory tests

Hematology	WBCs (neutrophils, lymphocytes, monocytes, eosinophils, basophils), RBCs, hemoglobin, hematocrit, platelets
Chemistry	Total cholesterol, triglyceride, total protein, albumin, A/G ratio, BUN, uric acid, creatinine, total bilirubin, AST (GOT), ALT (GPT), gamma-GTP, alkaline phosphatase, LDH, creatine kinase (CPK), calcium, phosphorus, sodium, potassium, chloride, CRP
Research specimen	8 mL of serum from 14-20 mL of blood
Urinalysis	Occult blood, glucose, protein

8-2-6. RT-PCR testing
Nasopharyngeal swab specimens will be collected for RT-PCR daily from Day 1 to Day 6, then on Days 8, 10, 12, 14 and 16 while the subject remains in hospital. In addition, results of RT-PCR performed as part of routine clinical care will also be recorded in the case report form, including the date of collection of the first of the two serial negative RT-PCR that were performed after enrollment.

8-2-7. Vital signs
The principal investigator or site investigator will record daily SpO_2, body temperature, systolic and diastolic blood pressures, pulse rate and respiratory rate from Day 1 to Day 15 or until study discontinuation. Data priory to enrollment should also be recorded in the case report form.
For SpO_2, the highest, lowest values and the value prior to administration (only on Day 1 for the early treatment group and Day 6 for the delayed treatment group) will be recorded. For body temperature, the highest axillary temperature and the temperature prior to administration (only on Day 1 for the early treatment group and Day 6 for the delayed treatment group) will be recorded in the case report form. For blood pressure, pulse rate and respiratory rate, the first measurements of the day will be recorded in the case report form.

8-2-8. Study drug administration
The principal investigator or site investigator will verify study drug administration on a daily basis and report the information, including time and route, in the case report form.

8-2-9. Duration of hospitalization
The subject will remain inpatient at least until the safety assessment on the day following the last dose of favipiravir. Assessment after hospital discharge will be performed as needed.

8-2-10. Assessment of pneumonia
Radiological assessment performed as clinically indicated, including presence or absence of pneumonia and associated findings, will be recorded in the case report form. Detailed assessment of pneumonia will be performed as a secondary study at a later time, in which images from clinically indicated radiological studies will be collected. Details will be stipulated in the protocol of the secondary study.

8-3. Risk management for the subjects
Favipiravir is known to cause increased uric acid levels. This is due to the effect of favipiravir and its major metabolite M1 on the transporter involved in renal excretion of uric acid, similar to that observed with pyrazinamide which is an antituberculosis drug. The increase of uric acid levels after favipiravir administration correlates with the cumulative dose, duration and exposure of favipiraivr. In the JP120 study conducted by FUJIFILM Toyama Chemical , in which healthy adults were given favipiravir 1,800 mg twice on Day 1, 800 mg twice a day from Day 2 to Day 21 and 800 mg once on Day 22, the uric acid levels increased linearly with increases of 2.7 mg/dL on Day 3, 5.5 mg/dL on Day 6 compared with before administration, then stabilized with an average increase of 6.3 mg/dL on Day 22. Discontinuation of favpiravir led to clearance of favipiravir and M1 from blood, resulting in an average uric acid level that was 3.4 mg/dL higher than before the administration two days after discontinuation and 0.4 mg/dL higher than before administration seven days after discontinuation. Among all study subjects who have received favipiravir thus far, gout and secondary acute kidney injury occurred in one subject who was being treated for a severe fever with thrombocytopenia syndrome (SFTS). The subject recovered after completion of favipiravir treatment. These findings suggest that favipiravir may be discontinued when gout occurs in association with increased uric acid levels. In particular, SARS-CoV-2 infection can cause dehydration from fever and other symptoms, which may make the patient prone to increased uric acid levels. Caution is therefore required when administering favipiravir to patients

with history of gout or hyperuricemia.
Additionally, in the JP120 study, 2 of 8 subjects experienced increased AST and ALT levels two weeks into administration of favipiravir and the ALT level increased to 137 IU/L two days after completion of the 22-day regimen, though these changes did not meet Hy's law. Alkaline phosphatase, gamma-GTP and total bilirubin levels were unchanged in these two subjects, and both AST and ALT normalized one week after completion of the regimen. Nonetheless, liver function tests should be monitored while receiving favipiravir.

8-3-1. Information on adverse events of favipiravir

The types and frequencies of adverse events associated with favipiravir use in clinical studies conducted in and outside Japan are listed in Table 3.
Serious adverse events for which association with favipiravir was not ruled out included hematochezia (1) and liver function abnormalities (2). No deaths or other serious adverse events have been observed.

Table 3. Adverse events reported to date

Type	≥1%	0.5-1%	<0.5%
Hypersensitivity		Rash	Eczema, pruritus
Hepatic	Increased AST (GOT), increased ALT (GPT), increased γ-GTP		Increased ALP, increased bilirubin
Gastrointestinal	Diarrhea (4.79%)	Nausea, vomiting, abdominal pain	Abdominal discomfort, duodenal ulcer, hematochezia, gastritis
Hematology	Neutropenia, Leukopenia		Leukocytosis, reticulocytopenia, monocytosis
Metabolism	Increased uric acid (4.79%), increased triglyceride	Glycosuria	Hypokalemia
Respiratory			Asthma, oropharyngeal pain, rhinitis, rhinopharyngitis
Others			Increased creatinine kinase, hematuria, tonsillar polyp, skin pigmentation, dysgeusia, abrasion, blurry vision, vertigo, premature supraventricular contraction

8-3-2. Potential direct benefits to the subjects
Favipiravir has shown efficacy against influenza, Ebola virus infection, and severe fever with thrombocytopenia syndrome (SFTS). It inhibits a replication of SARS-CoV-2 in vitro, which suggests potential clinical efficacy against COVID-19.

8-3-3. Risk mitigation
Subjects will remain inpatient until the day following the last dose of favipiravir.

9. Safety evaluation
9-1. Adverse events
Any unfavorable or unintended symptoms or signs (including abnormal laboratory values) that occur between the start and end of favipiravir administration will be considered adverse events regardless of causal relationship with favipiravir. However, progression of infection due to insufficient efficacy of favipiravir will not be considered an adverse event. Symptoms attributed to underlying illnesses or comorbidities are not considered adverse events, but they are considered as such if they worsen during favipiravir treatment, such as when an intervention is required.
If an adverse event occurs during the study period, the principal investigator or site investigator will investigate the nature of the event, date of event, grade, intervention, outcome, date of outcome, association with favipiravir, predictability of the event, impact on favipiravir treatment and record the information in the case report form.
If an adverse event leads to an illness, the subject will be followed until recovery from the illness, or until safety is ensured.

10. Criteria for evaluation
10-1. Adverse events
An adverse event will be classified to “Grade 1” through “Grade 5” in accordance with the five-point scale endorsed in JCOG/JSCO’s adverse event terminology criteria version 4.0 (Japanese) for evaluation of safety. If an event with an assigned grade cannot be identified, the grade will be determined based on the criteria for symptom severity as shown in Table 4.
Association of the event with favpiravir will be determined based on the criteria in Table 5.
Outcome will be determined based on the criteria shown in Table 6.

Table 4. Criteria for symptom severity

Category	Criteria
1. Mild	The subject can continue with the study; activity of daily living is not impaired
2. Moderate	Activity of daily living is impaired, but not completely
3. Severe	Activity of daily living is impaired due to due to permanent dysfunction; the subject requires medical intervention

Table 5. Criteria for association

Category	Criteria
1. Unrelated	Temporally unrelated to favipiravir, or the event is clearly unrelated to favipiravir
2. Related	Temporal association with favipiravir cannot be ruled out, or the event is not clearly unrelated to favipiravir

Table 6. Criteria for outcome

Category	Criteria
1. Resolved	Signs, symptoms, illnesses have resolved, normalized, or returned to baseline prior to administration
2. Improved	Signs, symptoms, illnesses have improved but have not resolved
3. Unchanged	Signs, symptoms, illnesses have not improved or worsened
4. Died	The subject died from the adverse event

10-2. Serious adverse events

In the event of a serious adverse event (see “Table 7. Serious adverse events”), the principal investigator or site investigator will determine whether favipiravir should be discontinued or not and provide appropriate treatment.

Table 7. Serious adverse events

1. Death
2. An event that may lead to death[a]
3. Disability
4. An event that may lead to disability
5. An event for which hospitalization or extension of hospitalization is required for management[b]
6. An event that is as serious as any of the above[c]
7. Congenital disease or abnormality in the offspring

[a] Defined as an event which poses risk of death at the time, not one that would pose risk of death were it more serious.

[b] Excludes hospitalization or its extension for the purpose of diagnostic tests, or for the purpose of observation after resolution or improvement of the adverse event. However, hospitalization or its extension to conduct significant medical care, such as surgery or mechanical ventilation, is included.

[c] An event that may lead to disability, or a serious adverse event that does not necessarily pose a threat of death or hospitalization but may necessitate intervention to prevent danger to the subject or outcomes as listed in 1-5.

10-2-1. Reporting to the Certified Review Board

When a site investigator learns of the following events, the site investigator will report it to the administrator representing the institution within the specified timeframe and notify the principal investigator. The principal investigator will expeditiously inform the site investigators at each participating institution of the events.

The site investigators at each participating institution will expeditiously report the information to the administrator representing the institution. The principal investigator will report to the administrator representing the institution and the certified review board.

A. Among the below events, those that may be caused by an unapproved or off-label medication during the course of the clinical study and are unexpected

7 days

1. Death
2. An event that may lead to death

B. Among the below events, those that may be caused by an unapproved or off-label medication during the course of the clinical study (excluding (A))

15 days

1. Death
2. An event that may lead to death

C. Among the below events, those that may be caused by an unapproved or off-label medication during the course of the clinical study and are unexpected (excluding (A))

15 days

1. An event for which hospitalization or its extension is needed for treatment
2. Disability
3. An event that may lead to disability
4. An event that is as serious as 1-3, death, or an event that may lead to death

5. Congenital disease or abnormality in the offspring

Events that may be related to the conduct of the clinical study are known, non-serious and not included in the above should be included in the regular report to the Certified Review Board.

10-2-2. Reporting to the Minister of Health, Labour and Welfare
The principal investigator will report the following events to the MHLW within the specified timeframe.

A. Among the below events, those that may be caused by an unapproved or off-label medication during the course of the clinical study and are unexpected
7 days
1. Death
2. An event that may lead to death

B. Among the below events, those that may be caused by an unapproved or off-label medication during the course of the clinical study and are unexpected
15 days
1. An event for which hospitalization or its extension is needed for treatment
2. Disability
3. An event that may lead to disability
4. An event that is as serious as 1-3, death, or an event that may lead to death
5. Congenital disease or abnormality in the offspring

10-2-3. Reporting to the provider of study drug
The principal investigator will notify the provider of the study drug upon learning about events that meet 10-2-1.

10-3. Reporting of pregnancy
The site investigators will direct subjects to report immediately if they become pregnant during study participation (28 days). Upon learning of possible pregnancy, the site investigator will expeditiously report the information in writing to the principal investigator. The site investigator will follow up on the outcome of pregnancy, including birth, spontaneous abortion, termination of pregnancy, and expeditiously report the outcome in writing to the principal investigator. Pregnancy outcomes including spontaneous abortion, termination of pregnancy, congenital anomaly, perinatal death will be considered serious adverse events. The site investigator will report such an event in writing, along with the report on the pregnancy outcome, to the principal investigator.

10-4. Provision of care upon completion of study participation
The site investigator must make all efforts to ensure the subject has access to the best prevention, diagnosis and treatment resulting from study participation even after completion of study participation.

11. Number of subjects
Early treatment group: 43 subjects
Delayed treatment group: 43 subjects
Total: 86 subjects
The computed number of subjects is based on the assumption that the virus will be undetectable by Day 6 in 80% of the subjects in the early treatment group and 50% of the subjects in the delayed treatment group. With power of ≥80% and a two-sided significance level of 0.05, the study requires 39 cases in each group. Accounting for a 10% drop-out rate, 43 subjects are required in each arm.
The computed number of subjects may be re-evaluated at the interim analysis which is planned when half the number of subjects are followed for the primary endpoint. The sample size may be

adjusted if the committee requests after examining the pooled (blinded for the study allocation) proportion of subjects reaching the primary endpoint. Changes in the numbers of subjects will be stipulated in the appendix as needed.

12. Study duration

From the date of jRCT registration through August 2020

13. Criteria for study discontinuation

The principal investigator and site investigators will withdraw subjects from the study and all study related treatments will be discontinued if any of the below conditions are met. When a decision of the withdrawal is made, laboratory tests should be performed and reasons for discontinuation will be documented in the source data. If a subject is still positive for SARS-CoV-2 by RT-PCR at the time of withdrawal, the investigator will document the outcome of the subject after the withdrawal, at least until the day following the discontinuation of favipiravir but as long as the investigators consider that it is necessary.

1. A subject or her/his legal representative requests to withdraw the subjects from the study
2. The principal investigator or site investigators determine to discontinue the study treatment due to adverse events
3. The principal investigator or site investigator determines the discontinuation to be appropriate due to insufficient efficacy of favipiravir or clinical deterioration
4. The subject is found to have been erroneously enrolled without meeting the inclusion criteria
5. The subject can no longer be followed
6. Continuation of the study is no longer appropriate by the judgment of the principal investigator or site investigators

14. Study discontinuation

14-1. Study completion

A site investigator will expeditiously report to the administrator of the institution and the principal investigator when the last study drug administration and observation as stipulated in the study protocol have been completed for the last subject at the institution.

14-2. Study discontinuation

The site investigator may decide to terminate or suspend the study if study continuation is not possible due to serious adverse events or other reasons.

15. Monitoring, auditing and data management

15-1. Monitoring and auditing

The site investigators will arrange for monitoring and audit, as necessary, to ensure that the study is conducted in compliance with the regulations, in adherence to the study protocol that was approved by the administrator of the institution, in order to ensure the reliability of the results.

The principal investigator will designate the monitors and auditors. The site investigator at each participating institution will delegate their appointments to the principal investigator.

The monitors and auditors will review the adequacy of the study process and accuracy of the study data and report the findings to the site investigator.

If the monitors encounter information that compromises study ethics or scientific integrity, or carries the risk of doing so, they will report it to the site investigator. The site investigator will address the concerns and report them to the administrator of the institution. The site investigator will also submit a report to the principal investigator, who will then notify the other site investigators of the findings.

The principal investigator and site investigator will review the findings of monitoring and amend the study protocol or procedures as appropriate. The detailed monitoring process will be stipulated in the monitoring protocol.

The auditors will generate an audit report and submit it to the site investigator at the institution

where the audit occurred. The auditors will also submit a copy of the report to the principal investigator.

15-2. Access to the source data

The site investigator will provide access to any source data related to the study, including contracts with the funding source, at the time of monitoring, audit, or any other investigations conducted by the Certified Review Board or regulatory bodies.

15-3. Data management

The site investigator will submit complete case report forms to the principal investigator. The principal investigator will assign a data manager. The data manager will conduct data management according to protocols.

15-4. Source data

Of the data contained in the case report forms, the documentation in the case report forms are considered source data for the below items.

- Race and nationality
- Presence of adverse events, their severity, need for intervention, date of outcome assessment, outcome, association with the study drug and other comments

16. Compliance to the study protocol

16-1. Compliance to the study protocol

The site investigators and co-investigators conduct the study in compliance with the Clinical Trials Act and the study protocol.

16-2. Deviation from the study protocol

If a deviation from the study protocol occurs, the site investigator or a co-investigator will address the deviation by recording the details, reporting them to the administrator of the participating institution and the principal investigator.
The principal investigator will inform the site investigators of the deviations that were reported from the site investigators.
In case the deviation is serious, the reporting should be expedited, and the principal investigator will seek feedback from the Certified Review Board over the serious deviation in question.

17. Statistical analysis

The statistical analysis plans are summarized below. Further technical and detailed items including statistical analysis plans for the primary variable, secondary variables and other data will be available in a separate Statistical Analysis Plan (SAP).

17-1. Analysis sets

Analysis sets for efficacy and safety will be defined as described below. Exclusion criteria are summarized along with the rationales. However, when it requires individualized decision on a subject exclusion, it will be discussed at the endpoint review committee meeting which will be blinded for study allocation. Analysis will be based on the intention-to-treat principle and consist of the following analysis sets. Efficacy endpoints will be assessed using the full analysis set (FAS). The primary endpoint will also be assessed using the per protocol set (PPS). Safety endpoints will be assessed using the safety analysis set (SAS).

The efficacy and safety analysis sets are defined as below.

1. Full analysis set (FAS)
   FAS consists of all subjects who enrolled into the study except those who meet any of the following criteria. The analysis will be conducted according to the assigned treatment group.

1. Subject for whom there is no efficacy data after the randomization
2. Subject whose Day 1 pre-dose PCR testing was already negative

2. Modified full analysis set (modified FAS)
   The modified FAS consists of all subjects who enrolled into the study except those who meet the below criterion. The analysis will be conducted according to the assigned treatment group.

   1. Subject for whom there is no efficacy data after randomization occurred

3. Per protocol set (PPS)
   The PPS consists of FAS after excluding those who have any of the major protocol violations listed below. If a subject drops out from the PPS during the course of study participation, the data immediately before that point of the protocol violation will be included in the PPS. The analysis will be conducted according to the assigned treatment group.

   1. Subject for whom a prohibited medication was used, or incorrect dosing of the study drug occurred
   2. For subject, whose study medication was discontinued or who withdrew from the study, data after the time of discontinuation or withdrawal from the study
   3. Subject who violated the inclusion or exclusion criteria

4. Safety analysis set (SAS)
   The SAS consists of all subjects after excluding those who meet the following criterion.

   1. Subject for whom no study medication was used

17-2. Efficacy analysis
Analysis set: FAS (primary), modified FAS (secondary)
Group: ITT

17-2-1. Primary efficacy endpoint
Proportion of subjects with clearance of SARS-CoV-2 by Day 6

Definition of SARS-CoV-2 clearance
Among subjects for whom SARS-CoV-2 was detected by RT-PCR from the Day 1 pre-dose nasopharyngeal swab specimen, the first day when SARS-CoV-2 was undetectable by RT-PCR from a nasopharyngeal specimen is defined as time to the viral clearance. Time to the viral clearance is defined as the day of clearance minus Day 1. For example, time to clearance is 1 day if clearance occurred on Day 2. The first day when SARS-CoV-2 was undetectable will be used as the day of clearance even if SARS-CoV-2 is later detected on a subsequent day.

Primary analysis
Using FAS, hazard ratios, their 95% confidence intervals and p-values will be calculated using the Cox proportional hazard regression model with time to SARS-CoV-2 clearance and the presence or absence of SARS-CoV-2 clearance by Day 6 as the dependent variables, treatment allocation as the explanatory variables, age (continuous variable) and time from the first positive RT-PCR test before the study enrollment (continuous variable) as covariates.
If a subject was discontinued before his/her RT-PCR results ever become negative, the subject will be censored on Day 6 or time of the discontinuation, whichever occurs earlier. If RT-PCR results are unavailable due to progression of the disease or death, an assumption will be made that SARS-CoV-2 clearance did not occur. Deaths which are unrelated to SARS-CoV-2 infection

will be censored on the day of death.

Secondary analysis

The cumulative proportion of SARS-CoV-2 clearance by Day 6 will be compared between the treatment groups with the stratified generalized Wilcoxon test. The stratifications will be made based on age (65 years or more vs less than 65 years) and time from the first positive RT-PCR test before enrollment (less than 7 days vs 8 days or more). If the number of subjects in each category (age, time from the first positive RT-PCR test before enrollment) is less than 10, then the subjects will be re-stratified based on the median age and the time from the first positive RT-PCR test before the enrollment.

In addition, the hazard ratio, its 95% confidence interval and p-values will be calculated using Cox proportional hazard regression models, with the time to SARS-CoV-2 clearance and the presence or absence of SARS-CoV-2 clearance by Day 6 as the dependent variables and the treatment assignment as the explanatory variable. Furthermore, Kaplan-Meier Product Limits estimators will be computed for each treatment group to calculate the median time to SARS-CoV-2 clearance and their 95% confidence intervals. The confidence intervals for proportions of SARS-CoV-2 clearance will be calculated by the Greenwood method, and the confidence intervals for the median days will be calculated by the Brookmeyer-Crowley method. Restricted Mean Survival Time (RMST) method will be used to estimate the mean number of days till SARS-CoV-2 clearance by Day 6 and its 95% confidence interval will be calculated.

If SARS-CoV-2 clearance does not occur by Day 6 or earlier, the subjects will be censored either on Day 6 or on the date of the last RT-PCR, whichever occurs earlier. If RT-PCT results are unavailable due to disease progression or death, an assumption will be made that SARS-CoV-2 clearance did not occur by Day 6. Deaths unrelated to SARS-CoV-2 will be censored on the day of death.

As an exploratory subgroup analyses, subgroups will be generated based on the initial SARS-CoV-2 viral load, the presence or absence of symptoms, age 65 and greater or less, time from the first positive RT-PCR result prior to study enrollment to examine if efficacy is observed in these subgroups. The threshold value of SARS-CoV-2 viral load for stratification will be defined prior to data lock.

The above analyses on FAS excludes subjects who had an undetectable RT-PCR result on Day 1. To assess the robustness of results when these excluded subjects were added to the analysis, proportions of SARS-CoV-2 clearance between the treatment groups will be compared in the modified FAS. However, since the survival time analysis used in the primary analysis cannot be applied to subjects with an undetectable RT-PCR result on Day 1, the modified FAS will be subjected to the following analyses examining whether SARS-CoV-2 clearance was achieved by Day 6 or not, using the binary logistic regression with adjustment for age (continuous variable) and time from the first positive RT-PCR test before the study enrollment (continuous variable) as covariates. Data for subjects whose last RT-PCR test occurred before Day 6 without SARS-CoV-2 clearance for reasons other than disease progression will be excluded.

17-2-2. Secondary efficacy endpoints

1. **SARS-CoV-2 clearance by Day 10 in FAS and the modified FAS**

   A similar analytical method described for the viral clearance by Day 6 will be used. If the last available RT-PCR result occurs without SARS-CoV-2 clearance on Day 10 or earlier, the results will be censored on the day of the last available RT-PCT or Day 10, whichever is earlier. If RT-PCR results are unavailable due to disease progression or death, an assumption will be made that the subject was followed through Day 10 and SARS-CoV-2 clearance did not occur. Subjects will be censored at the time of death if death was unrelated to SARS-CoV-2.

2. **50% logarithmic reduction in the SARS-CoV-2 viral load in FAS**

Whether reduction in the SARS-CoV-2 viral load of 50% or greater in the logarithmic scale is achieved on Day 6 and Day 10 compared with Day 1 will be assessed as defined below. For subjects in which RT-PCR results were unavailable due to disease progression or death, they will be considered as not having the 50% viral reduction.
If subjects are considered as not having RT-PCR and the reason of the lack of RT-PCR were due to disease progression or death unrelated to SARS-CoV-2, they will be censored on the day of disease progression. Those related to SARS-CoV-2 will be considered as no reduction in the SARS-CoV-2 viral load.

Logarithmic viral load reduction (%)
=[(logarithmic viral load on Day 1 pre-dose - logarithmic viral load on Day 6 or Day 10)/(logarithmic viral load on Day 1 pre-dose – logarithmic limit of detection)]x100

In addition, the viral load on Day 6 will also be considered below the limit of detection if it is achieved before Day 6. Likewise, the viral load on Day 10 will also be considered below the limit of detection if it is achieved before Day 10.

The odds ratio, its 95% confidence interval, p-values for will be calculated in FAS using 50% logarithmic reductions in SARS-CoV-2 viral load as the dependent variables, treatment groups as explanatory variables, age (continuous variable) and time from the first positive RT-PCR result prior to enrollment (continuous variable), as covariates in a binomial logistic regression model.

3. **Changes in the logarithmic SARS-CoV-2 viral load in FAS and modified FAS**

Average of logarithmic SARS-CoV-2 viral load values with a base of 10 will be compared between the treatment groups for each timepoint. When the viral load is below the limit of detection, it will be assigned to the value of 120 before logarithmic transformation. The data will be summarized as the logarithmic SARS-CoV-2 viral load for each treatment group at each timepoint, changes from Day 1 measured as mean and median values, standard deviations, interquartile ranges (25 percentile-75 percentile), and ranges (minimum-maximum).
The mixed effect regression model (MIXED model) will be used for the comparison between the treatment groups. In the MIXED model, the SARS-CoV-2 viral load values for each day of observation will be compared between the treatment groups using the logarithmic viral loads for each day as the dependent variables, logarithmic viral load values at baseline, age (continuous variable), time from the first positive RT-PCR result prior to enrollment (continuous variable) as covariates, Days (categorical variable), treatment assignment variable, a cross-product term of Days and treatment assignment as the explanatory variables, and using LSMEANS option. The normality of residuals will be assessed, and if absent, mathematical transformation of the dependent variables will be attempted to normalize the residuals. Deaths unrelated to SARS-CoV-2 will be censored on the days of death. Transition of mean (and 95% CI) viral loads across all time points will be graphically presented for each group.

4. **Time until SARS-CoV-2 clearance by non-study, mandatory RT-PCR testing**

Time from the start of the study to SARS-CoV-2 clearance by RT-PCR using nasopharyngeal or pharyngeal swab specimens performed at the participating institution for the purpose of meeting the government-sanctioned discharge criteria (i.e. two serial negative RT-PCR results) will be evaluated. The event occurrence day is defined as the day on which the first of the two negative RT-PCR results was collected. The time till the endpoint is calculated as the date of the event occurrence subtracted by the date of Day 1 pre-dose. If the follow-up was discontinued within 28 days and the event is not observed in this period, the subject will be censored on the day when the last RT-PCR is collected.

If the first of the two serial negative RT-PCR results is not obtained by Day 28 and the follow-up was made until the Day 28, the subject will be censored on Day 28. If the subject dies before Day 28 due to progression of disease, the subject is considered to have been followed through Day 28 without negative RT-PCR results, subjects will be censored on Day 28. Deaths unrelated to SARS-CoV-2 will be censored on the day of death.
The hazard ratio, its 95% confidence interval and p-value will be calculated in FAS using time to SARS-CoV-2 clearance by Cox proportional hazard model using non-study RT-PCR testing as the dependent variables, treatment groups as the explanatory variables, age and time from the first positive RT-PCR result prior to enrollment as covariates.
The same logistic regression analysis as described in 17-2-1 will be conducted in the modified FAS.

17-2-3. Exploratory efficacy endpoints

1. **Duration of fever**

Duration of fever will be analyzed in the modified FAS. Defervescence will be defined as body temperature of less than 37.5°C and less than 37.0°C. The hazard ratio, its 95% confidence interval and p-value will be calculated using time from Day 1 to the day of defervescence as the response variables, the treatment groups as the explanatory valuables, and age and time from the first positive PCR result prior to enrollment as covariates by the Cox proportional hazard model. Median time to defervescence and its 95% confidence intervals will be calculated using Kaplan-Meier curves for each group. The confidence intervals will be obtained by the Brookmeyer-Crowley method. The analysis will be conducted in the same manner as that of the primary efficacy endpoint (time to SARS-CoV-2 clearance), with the exception that subjects who were afebrile on Day 1 pre-dose will be excluded.

2. **Change in body temperature**

Changes in body temperature will be assessed in a combination of the following two analysis-sets using the following two dependent variables.

Group 1: All subjects in the modified FAS except those who did not have fever on Day 1 pre-dose
Group 2: All subjects in the modified FAS

Dependent variable 1: Presence or absence of body temperature of 37.5°C or greater or 37.5°C or greater on each day
Dependent variable 2: Actual body temperatures on each day

The proportions of subjects with body temperature of 37.5°C or greater on each day of observation will be compared between the treatment groups. The results will be summarized as proportions and frequencies of subjects with fever in each group on each day of observation. The inter-group comparison for dependent variable 1 will be conducted by the regression model using generalized estimating equation (GEE). That of dependent variable 2 will be conducted by the mixed effect model. Estimated regression coefficients, their 95% confidence intervals and p-values will be calculated by the regression model using the treatment groups as the explanatory variables, and age and time from the first positive PCR result prior to enrollment as the covariates.

In case the number of events is not sufficient, binomial logistic regression, the chi square method or Fisher’s exact method will be used.
The mean body temperature recorded on each day of observation from the start to the end of the study will be compared between the groups. The results will be summarized as the day of observation, mean body temperatures in each group, standard deviations, medians, interquartile ranges (25 percentile to 75 percentile), and ranges (minimum to

maximum).

3. **Change in symptoms**
   The symptom scores (entire scores and each of the four components of the scores) will be compared on each day of observation from the start to the end of the study in FAS and modified FAS. The results will be summarized as the day of observation, mean symptom scores in each group, standard deviations, medians, interquartile ranges (25 percentile to 75 percentile), and ranges (minimum to maximum). The inter-group comparisons will be adjusted using stratification variables at randomization as covariates by the GEE regression model.

4. **Disease progression and death**
   The hazard ratio, its 95% confidence interval and p-values will be calculated with time to death, mechanical ventilation or ICU admission as the response variables, treatment groups as the explanatory variables, age and time from the first positive RT-PCR test before enrollment as covariates by the Cox proportional hazard model.
   Median time to any of these three events and its 95% confidence intervals will be calculated using Kaplan-Meier curves for each group. The confidence intervals will be obtained by the Brookmeyer-Crowley method. The analysis will be conducted in the same manner as that for the primary efficacy endpoint. If the number of events is too small for inferential statistics, only descriptive statistics will be summarized.

5. **Death**
   The hazard ratio, its 95% confidence interval and p-values will be calculated with time to death as the response variable, treatment groups as the explanatory variables, age and time from the first positive RT-PCR test before enrollment as covariates by the Cox proportional hazard model.
   Median time to death and its 95% confidence intervals will be calculated using Kaplan-Meier curves for each group. The confidence intervals will be obtained by the Brookmeyer-Crowley method. The analysis will be conducted in the same manner as that for the primary efficacy endpoint. If the number of events is too small for inferential statistics, only descriptive statistics will be summarized.

Examination of the robustness of the regression models
For each of the regression analyses described above, analysis without adjustment for the stratification variables (age and time from the first positive PCR result prior to enrollment) as covariates will also be conducted to assess their robustness. When a meaningful difference is observed on a baseline variable, as a post-hoc analysis, the baseline variable will be added to the regression models to the model with the two stratification variables.

Interim analysis
An interim analysis will be performed on the primary efficacy endpoint to assess compliance to the study protocol and safety. The analysis will occur once the data on the primary efficacy endpoint (time to SARS-CoV-2 clearance) have been accrued for at least 22 subjects in each treatment group. Since efficacy and safety will be assessed twice during the course of the study, two-sided p-value of 0.001 and 0.05 will be adopted for the interim and final analyses, respectively, in accordance with the Haybittle-Peto rule. If the estimated final event frequency between the treatment groups falls below 56 in FAS at the interim analysis under the assumption that the dropout rate and event frequency remain the same before and after the interim analysis, the enrollment target may be adjusted so that the final event frequency is expected to reach 56, if it is permitted by the independent data monitoring committee (IDMC). The principal investigator will only be notified of the interim analysis results in aggregate and not by treatment groups. The unblinded results will be reported to the IDMC only.

Interim analysis of secondary efficacy endpoints and other items will be conducted if the IDMC determines that a review of the data is necessary. Whether the interim analysis results should be announced publicly will be determined by the IDMC.

17-3. Safety analysis

Adverse events

The numbers of subjects and incidents for each adverse event will be calculated for each treatment group. They will also be calculated based on severity of illness and possibility or probability of association with the study drug.

Other safety items

Vital signs, laboratory testing results will be summarized for each treatment group as appropriate.

17-4. Others

Unless specified, all tests will be based on two-sided hypotheses with a significance level of 5%. For data derived from observations across multiple days, such as time to SARS-CoV-2 clearance or duration of fever, missing data points after the final observation will not be imputed, but those before the final observation will be imputed using the last-observation-carried forward (LOCF) method. For example, if virus is detected on Day 2, the data are missing on Day 3, and the virus is undetectable on Day 4, the missing data on Day 3 will be imputed by the Day 2 data. Additionally, robustness of the analysis will be examined without imputations in case data in the explanatory variables of regression models are missing.

18. Compensation

If health damage occurs to the subjects due to their participation to this study, the participating institution will provide necessary care and the site investigator will report the incident to the principal investigator.

Compensation will be provided to the subjects for health damage according to the insurance contract with the insurance company providing coverage for the study. However, compensation will not be provided for health damage for which association with the study drug can reasonably be ruled out, health damage due to progression of underlying illnesses, or lack of anticipated clinical benefit from the study drug or other benefits.

19. Secondary use of study data

The data obtained through this study may be shared with the MHLW and the National Institutes of Health for use in further research on this disease or development of new therapeutic modalities. In addition, the interim and final analysis reports may be disclosed to FUJIFILM Toyama Chemical at the request from the government for the sole purpose of including the data in a new drug application. The data will be deidentified for protection of personal information.

20. Management and disposal of study data, specimens

Specimens obtained through this study will be rid of personal identifiers and disposed of after the conclusion of the study.

The site investigators will maintain the documents and records related to this study for five years from the day the closure of the study is announced.

21. Registration of study plan and announcement of study data

This study will be registered to the Japan Registry of Clinical Trials (jRCT) administered by the MHLW prior to the start. The contents in the jRCT will be updated as appropriate to reflect revisions of the study protocol and progress of the study. The final results will be posted at the conclusion of the study in a timely manner.

The interim and final analysis reports may be disclosed to FUJIFILM Toyama Chemical only for the purpose of their use in a new drug application of favipiravir at the request of the government. (This clause was added in response to the following notification from the MHLW: “Handling of

approval review of medical products intended for the new coronavirus infection" issued on May 12, 2020.)

22. Funding and conflicts of interest

The study will be funded by the Japan Agency for Medical Research and Development (AMED). Favipiravir used in the study will be donated by FUJIFILM Toyama Chemical under a contract. FUJIFILM Toyama Chemical will supply information regarding the study drug but will not be involved in the conduct of the study.
Conflicts of interest will be managed appropriately according to the guidance for management of conflicts of interest. None of the principal investigator, site investigators, or co-investigators had any conflicts of interest to declare with FUJIFILM Toyama Chemical.

23. Costs to subjects

Favipiravir will be donated by FUJIFILM Toyama Chemical, its manufacturer. The medical costs during study participation will not be borne by the study in principal. However, some of the costs may be borne by a participating institution at its discretion. The subjects will not be compensated financially for their participation in the study.

24. Management of protected health information

The materials and documents related to the study will be stored securely for the designated storage periods at the site investigator's responsibility at each participating institution. Electronic data will be password-protected, and paper documents will be locked in cabinets. Once the designated storage periods have passed, the materials will be disposed of upon notification from the principal investigator in a manner that is unrestorable.
Specimens will be anonymized of personal information (such as name, address and phone number) and identified by subject IDs. The subject IDs will also be used in exchanging data among the participating institutions, principal investigator, central laboratory, clinical research organizations and others that may be involved in the study. Personal information of the subjects will not be disclosed in any abstracts or manuscripts that may result from the study, or in any secondary studies.

25. Reporting to participating institutions and Minister of Health, Labour and Welfare

The principal investigator, site investigators and co-investigators will comply with the Clinical Trials Act (Act No. 16 of April 14, 2017) and report to the administrators of the participating institutions and the MHLW as appropriate.

26. Consultation from subjects and their legal representatives

The contact information will be listed in the consent form for the subjects and their legal representatives.

27. Study organization

The study organization will be listed in the study organization document.

28. Contracting

The contracting parties and contents will be listed in the study organization document.

29. References

1 Gowen BB, Wong MH, Jung KH, Sanders AB, Mendenhall M, Bailey KW, et al. In vitro and in vivo activities of T-705 against arenavirus and bunyavirus infections. Antimicrob Agents Chemother. 2007;51:3168-76.
2 Mendenhall M, Russell A, Smee DF, Hall JO, Skirpstunas R, Furuta Y, et al. Effective oral favipiravir (T-705) therapy initiated after the onset of clinical disease in a model of arenavirus hemorrhagic Fever. PLoS Negl Trop Dis. 2011;5:e1342

3 Oestereich L, Lüdtke A, Wurr S, Rieger T, Rieger T, Muñoz-Fontela C, et al. Successful treatment of advanced Ebola virus infection with T-705 (favipiravir) in a small animal model. Antiviral Res. 2014;105:17-21.

Multicenter, open-label, randomized trial of favipiravir in asymptomatic and mildly symptomatic patients infected with SARS-CoV-2 to evaluate viral load reduction

Participating institutions

Fujita Health University Hospital
Tokyo Metropolitan Health and Hospitals Corporation Ebara Hospital
Nara Medical University Hospital
St. Marianna University School of Medicine Hospital
Fujita Health University Bantane Hospital
National Hospital Organization Hokkaido Medical Center
Asahikawa City Hospital
Sagamihara Kyodo Hospital
Japanese Red Cross Nagoya Daiichi Hospital
Yokohama City University Hospital
Tosei General Hospital
Okazaki Municipal Aichi Hospital
Ehime Prefectural Central Hospital
Shimonoseki City Hospital
Kyorin University Hospital
National Hospital Organization Kanazawa Medical Center
Japanese Red Cross Narita Hospital
Saiseikai Niigata Hospital
Tottori Prefectural Kousei Hospital
Sapporo Medical Center, Nippon Telegraph and Telephone
Japanese Red Cross Ishinomaki Hospital
Isehara Kyodo Hospital
Minami-Nara General Medical Center
Shimane University Hospital
Kasugai Municipal Hospital
Yao General Hospital
Kagoshima City Hospital
Okayama City Hospital
Tokyo Shinagawa Hospital
National Hospital Organization Nagoya Medical Center
Kanto Rosai Hospital
Hamamatsu Medical Center
St. Luke's International Hospital
University Hospital Kyoto Prefectural University of Medicine
Kagawa Prefectural Central Hospital
Nozaki Tokushukai Hospital
Aomori Prefectural Central Hospital
International University of Health and Welfare Narita Hospital
Juntendo University Hospital
Ishikawa Prefectural Central Hospital
Fukuoka Tokushukai Hospital
Shonan Kamakura General Hospital
Komatsu Municipal Hospital
Ome Municipal General Hospital
Kobe City Medical Center General Hospital
Kitakyushu Municipal Medical Center
Eiju General Hospital
